# *Cryptocarya alba* (Peumo): an endemic Chilean tree with phytochemicals with bioactive potential

**DOI:** 10.3389/fphar.2025.1665897

**Published:** 2025-12-03

**Authors:** Gonzalo Fuentes-Barros, Sebastián Castro-Saavedra, Nicolás Montalva, Marco Mellado, Antonia Diaz-Valdés, Claudia Guerrero-Rodríguez, Javier Echeverría

**Affiliations:** 1 SAPHYCHEM, Santiago, Chile; 2 Programa de Doctorado en Políticas Públicas, Universidad Mayor, Santiago, Chile; 3 Departamento de Ciencias del Ambiente, Facultad de Química y Biología, Universidad de Santiago de Chile, Santiago, Chile; 4 Centro de Investigación en Sociedad y Salud, Facultad de Ciencias Sociales y Artes, Universidad Mayor, Santiago, Chile; 5 Centro de Investigación en Ingeniería de Materiales, Universidad Central de Chile, Santiago, Chile; 6 Facultad de Ciencias Sociales y Artes, Psicología, Universidad Mayor, Santiago, Chile; 7 Centro de Gerociencia para la Salud Cerebral y el Metabolismo, Santiago, Chile

**Keywords:** cryptocarya genus, Cryptocarya alba, peumo, phytochemistry, alkaloids, essential oils, phenolic compounds, pharmacology

## Abstract

**Background:**

*Cryptocarya alba* (Mol.) Looser [Lauraceae], known as *peumo*, is an endemic species of the central Chilean landscape. *C. alba* has an essential ecological role in the threatened sclerophyllous forest, with traditional uses of leaves, bark, and fruits, and the biotechnological and pharmacological potential of its phytochemicals.

**Purpose:**

The aim is to present the first comprehensive review of the current state of knowledge regarding traditional uses, ethnopharmacology, chemical composition, pharmacokinetic profile, and biological activities of *C. alba*.

**Methodology:**

Literature data on the traditional uses, ethnopharmacology, chemistry, and bioactivity of *C. alba* were primarily obtained from digital databases, including Scopus®, ScienceDirect®, SciFinder®, PubMed®, SciELO, and Google Scholar®, as well as from the scientific journal publishers’ platforms associated with these databases.

**Results and discussion:**

Traditional uses include its role as a food source for prehistoric populations and ethnomedicinal applications for liver diseases, rheumatism, and infections. The aerial parts are rich in polyphenols, including chlorogenic acid, epicatechin, procyanidins, quercitrin, rutin, and hyperoside, as well as essential oils derived from the leaves. While it contains various alkaloids, only reticuline is present in significant amounts, contributing to the species' highly variable chemical composition. Studies evaluating the biological and pharmacological properties of its extracts and constituents are limited to a few *in vitro* and *in vivo* studies; to date, no preliminary or clinical studies are available.

**Conclusion:**

The review highlights the entire existing ethnographic and cultural context of *C. alba*, revealing a significant gap in information about the species. Although there is a strong historical component, it supports the bioactivity of its main secondary metabolites, given its chemical and pharmacological profile. Given the limited nature of current biological and pharmacological evaluation studies, future research should focus on advancing preclinical and clinical trials, as well as toxicology studies, to ensure the safe and effective use of this approach.

## 1 Introduction

The Lauraceae family is widely distributed in tropical and subtropical regions, particularly in the forests of Asia and the Americas. Comprising about sixty-seven genera and more than 2,500 species, this plant family plays a vital role in biodiversity within many ecosystems, and its significant contribution to floral diversity is evident in various forests (Custodio and da Veiga Junior, 2014). Many species in this family have been used to produce valuable products across multiple industries, including food, timber, pharmaceuticals, traditional medicine, and perfumery. This diverse application is due to the presence of fragrant and bioactive compounds that offer medicinal, antioxidant, antimicrobial, aromatic, and sensory properties (Custodio and da Veiga Junior, 2014). The *Cryptocarya* genus [Lauraceae], with more than 300 species worldwide, is known for thriving in mountain environments across the tropics (van der Merwe et al., 2016). Human use of these species is evident through the utilization of their biomass. Archaeological remains of *Cryptocarya liebertiana* and *C. wyliei* charcoal found at hearths in the Sibudu site in South Africa suggest that their wood was burned to produce aromatic smoke and that their fragrant leaves served as insect repellents approximately 58,000 years ago (Lennox, 2019). However, the genus continues to present taxonomic challenges due to differing interpretations of its characteristics (Huang et al., 2025).

*Cryptocarya alba* (Mol.) Looser, commonly known as peumo, penu, pegu, or pengu in the Mapuche language, is a tree species endemic to Chile in the Lauraceae family. This shade- and frost-tolerant evergreen tree grows between 30° and 40°S, reaching altitudes of up to 1,500 m above sea level (Fuentes-Ramírez et al., 2011). Its habitat includes both the Mediterranean climate zone of Chile and the northernmost part of the rainy temperate region, making it the southernmost member of its genus (Benoit Contesse, 1989). The species is abundant in central Chile, where it is a dominant component of the sclerophyllous forest, alongside *Peumus boldus* Mol. [Monimiaceae], *Lithraea caustica* Mol. [Anacardiaceae], and *Quillaja saponaria* Mol. [Quillajaceae]. The area where *C. alba* is found is recognized as one of the twenty-five biodiversity hotspots worldwide due to its high number of endemic species (Myers et al., 2000). The Mediterranean region of Chile is one of five global enclaves characterized by this climate type and is renowned for its exceptional floral diversity (Fuentes-Castillo et al., 2019). These five Mediterranean-climate regions have been key areas for human and biological evolution for thousands of years (Rick et al., 2020).

Despite its importance, the sclerophyllous forest is currently facing severe impacts (Miranda et al., 2017). In particular, *C. alba* faces significant threats due to habitat destruction and land-use change (Fuentes-Ramírez et al., 2011). These threats have led to its classification as a vulnerable species in some areas of central Chile, although it receives greater protection in the southernmost part of its range (Benoit Contesse, 1989). As deforestation and pressures on natural resources increase, evaluating the medicinal properties and chemical uses of plants has become a key strategy for conserving ecologically rich and highly endemic species, especially in Chile’s Central biodiversity hotspot (300,000 km^2^) (Myers et al., 2000). These studies not only protect the natural and cultural heritages associated with these species but also promote a sustainable economy through responsible plant cultivation and harvesting, providing a viable alternative to mitigate deforestation (Mølgaard et al., 2011).

Therefore, this article aims to provide the first comprehensive review of current knowledge on the traditional uses, phytochemistry, biological and pharmacological activities of *C. alba*, highlighting its unique potential, clarifying its bioactive properties, and identifying new research opportunities.

## 2 Methodology

Literature data on the traditional uses, ethnopharmacology, chemistry, and bioactivity of *C*. *alba* were primarily gathered from digital databases, including Scopus®, ScienceDirect®, SciFinder®, PubMed®, SciELO, and Google Scholar®, as well as from the platforms of scientific journal publishers linked to these databases. The search incorporated the following keywords: (“cryptocarya alba” OR “peumo”) AND (“medicinal plants” OR “ethnomedicine” OR “archaeology”). Additionally, the keywords “reticuline,” “chlorogenic acid,” “epicatechin,” and “procyanidin” were included. All peer-reviewed journal publications up to June 2025 were reviewed. Journal eligibility was based on articles written in English and Spanish. The inclusion criteria were established based on the proposed inquiries outlined in the introduction: i) traditional uses, ii) ethnopharmacology, iii) chemistry, and iv) *in vitro* and *in vivo* bioactivity. Exclusion criteria were studies that did not specifically address the composition, uses, and effects of *C. alba*. The chemical compounds in the biomass were classified by pathway and superclass using the NPClassifier tool (Kim et al., 2021). A structural and property similarity analysis was performed on the compounds described in the literature. Compounds with highly correlated characteristics were grouped to simplify analysis and comparison. To ensure accuracy and completeness, whenever a compound’s description was ambiguous, incomplete, or inconsistent, direct contact was made with the researchers responsible for the original publication to obtain clarifications and additional data. The Administration, Distribution, Metabolism, and Excretion (ADME) properties of the secondary metabolites present in *C*. *alba* were calculated using the SwissADME platform (Daina et al., 2017).

## 3 Phytochemistry

The phytochemical study of *C. alba* biomass has recently increased significantly due to its high and diverse content of secondary metabolites (Giordano et al., 2019; Peña-Rojas et al., 2021). In the literature review, a total of 211 metabolites have been identified for this species. Among them, 49 polyphenolic compounds were reported, including flavonoids (*n* = 22), cinnamic acid derivatives (*n* = 8), anthocyanins (*n* = 4), proanthocyanidins (*n* = 3), benzoic acid derivatives (*n* = 2), catechin (*n* = 1), epicatechin (*n* = 1), and miscellaneous polyphenols compounds (*n* = 8). Additionally, 15 alkaloids have been documented, which are distributed among aporphines (*n* = 8), benzylisoquinolines (*n* = 5), and isoquinolines (*n* = 2). Furthermore, essential oils (EOs) composition studies reported the presence of 147 constituents, distributed as 62 monoterpenes [acyclics (*n* = 14), monocyclics (*n* = 29), and bicyclics (*n* = 19)], 38 sesquiterpenes [acyclics (*n* = 4), monocyclics (*n* = 7), bicyclics (*n* = 17), and tricyclics (*n* = 10)], and miscellaneous compounds (*n* = 47).

### 3.1 Phytochemistry of aerial parts of *Cryptocarya alba*

#### 3.1.1 Polyphenols in the aerial parts of *Cryptocarya alba*

The growing interest in identifying natural antioxidants has led to the investigation of plant species as sources, with attention to the entire aboveground biomass (Wan et al., 2023). Polyphenols are chemical compounds found in different plants; they are known to have a wide range of health benefits, including protective effects on the liver and cardiovascular system (Yeung et al., 2021). These compounds have shown promise in managing non-communicable chronic diseases (NCCDs) due to their multiple health-promoting properties (Lorca et al., 2025). They serve as multitargeted therapeutic agents with pharmacological activities that include anti-inflammatory, antioxidant, and neurotrophic effects (Kooshki et al., 2023).

The initial study on polyphenols in 2.5 kg of air-dried leaves and stems from *C. alba* identified isorhamnetin (22 mg), kaempferol (23 mg), quercetin (32 mg), isorhamnetin-3-*O*-rhamnoside (36 mg), isorhamnetin-3-*O*-galactoside (26 mg), isorhamnetin-3-*O*-glucoside (30 mg), kaempferol-3-*O*-galactoside (72 mg), quercetin-3-*O*-rhamnoside (quercitrin) (204 mg), quercetin-3-*O*-galactoside (hyperoside) (121 mg), quercetin-3-*O*-glucoside (isoquercitrin) (173 mg), and chlorogenic acid (128 mg) (Timmermann et al., 1995).

Recent advances in phytochemistry, phytotherapy, and related fields exploring the medicinal use of plants have renewed interest in these resources, leading to new research and therapeutic applications (Salmerón-Manzano et al., 2020). Using high-performance liquid chromatography-diode-array detection-mass spectrometry (HPLC-DAD-MS) analysis, researchers identified chlorogenic acid, hyperoside, quercetin-3-*O*-pentoside, and kaempferol 3-*O*-glucoside as the primary compounds in the aerial parts of the plants. Smaller amounts of luteolin-8-*C*-glucoside (orientin), isoquercitrin, and apigenin-8-*C*-glucoside (vitexin) were also detected (Simirgiotis, 2013). An extract from *C. alba* leaves collected in southern Chile was standardized as rich in quercitrin, chlorogenic acid, kaempferol-3-*O*-β-galactoside according to (Timmermann et al., 1995), myricetin, *p*-coumaric acid, and rutin. The most abundant anthocyanins were cyanidin, peonidin, and malvidin (Carmona et al., 2017). In young branches, a chemical profile similar to that found in the leaves was observed, but at 90% lower concentration. This material showed a strong dominance of catechin, epicatechin, procyanidins, protocatechuic acid, and vanillinic acid (Peña-Rojas et al., 2021).

Various catechin monomers and dimers have been identified in the bark of *C. alba*. These compounds include epigallocatechin-catechin dimer (*m/z* 594), catechin, epicatechin, procyanidin B1, procyanidin B2, and procyanidin C1 (Simirgiotis, 2013; Giordano et al., 2019; Antileo-Laurie et al., 2023).

**FIGURE 1:**
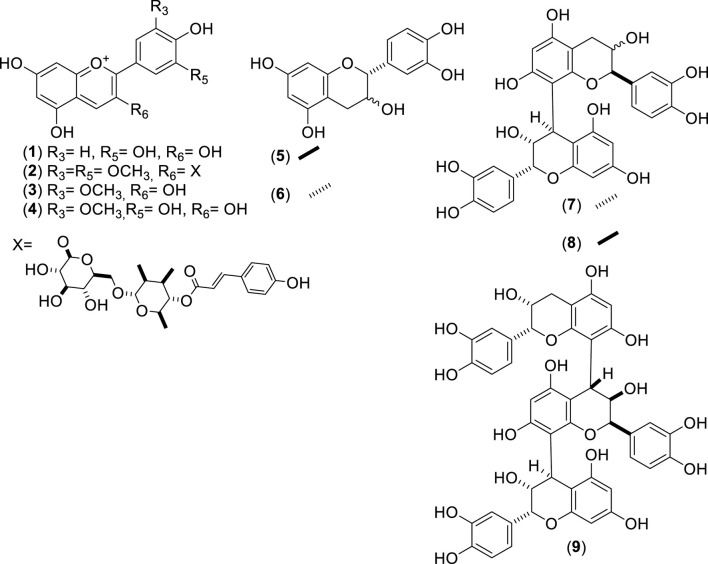
Chemical structures of anthocyanins (ACNs, 1–4), catechins (CATs, 5–6), and procyanidins (PCNs, 7–9) identified from *Cryptocarya alba*.

**FIGURE 2:**
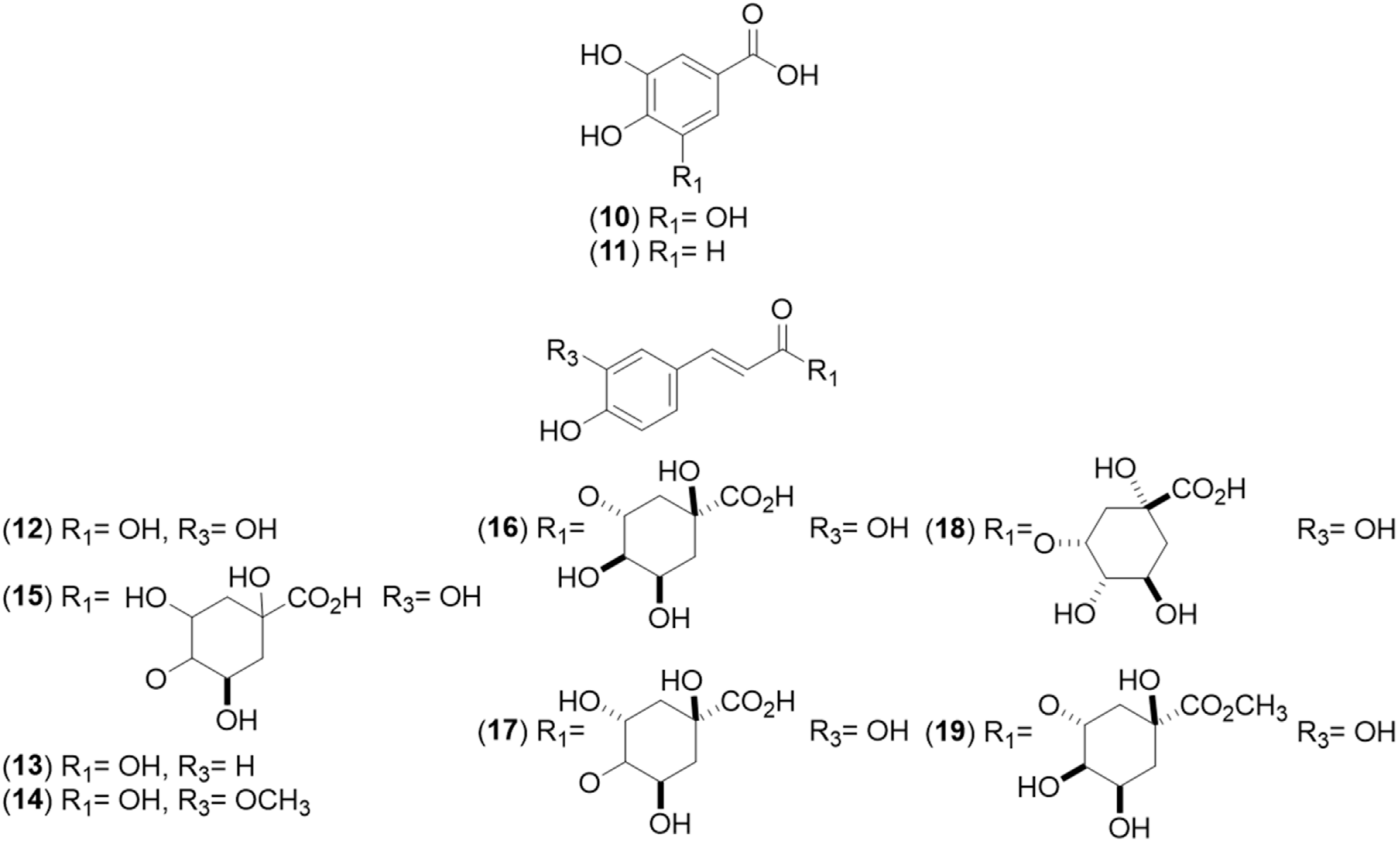
Chemical structures of benzoic (BAs, 10–11) and cinnamic acid (CAs, 12–19) derivatives identified from *Cryptocarya alba*.

**FIGURE 3:**
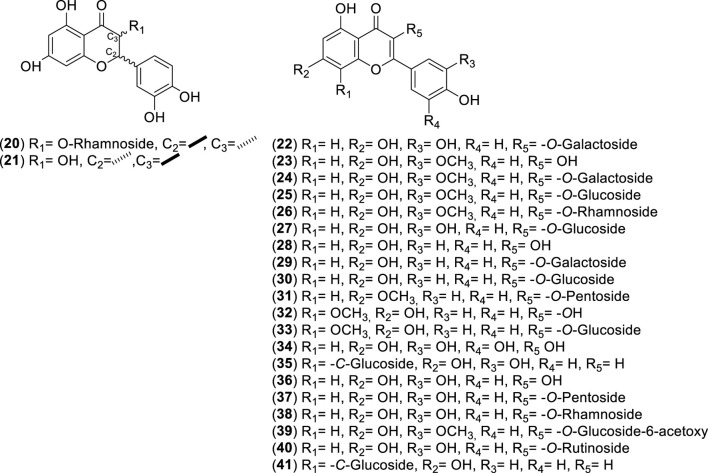
Chemical structures of flavonoids (Fs, 20–41) identified from *Cryptocarya alba*.

**FIGURE 4:**
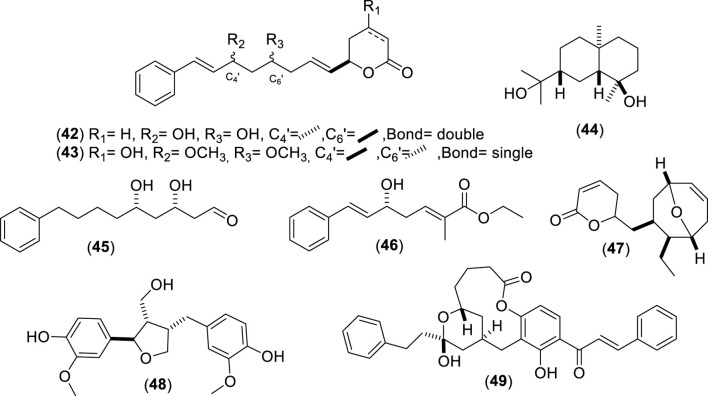
Chemical structures of miscellaneous phenolic compounds (MPCs, 42–49) identified from *Cryptocarya alba*.

The phenolic compounds found in *C. alba* are listed in Table 1, and structures are provided in Figures 1–4.

**TABLE 1:** Phenolic compounds identified in *Cryptocarya alba*.

n°	Compound	Part	Pathway	Superclass	Class	Identification	References
1	Cyanidin	L	Shikimates and Phenylpropanoids	Flavonoids	Anthocyanidins	HPLC–DAD + RS	Carmona et al. (2017)
2	Malvidin-3-*O*-(4‴coumaroyl)-rutinose	L	Shikimates and Phenylpropanoids	Flavonoids	Flavonoids	LC-DAD + LC-MS + MS/MS	Simirgiotis (2013)
3	Peonidin	L	Shikimates and Phenylpropanoids	Flavonoids	Anthocyanidins	HPLC–DAD + RS	Carmona et al. (2017)
4	Petunidin	L	Shikimates and Phenylpropanoids	Flavonoids	Anthocyanidins	HPLC–DAD + RS	Carmona et al. (2017)
5	Catechin	L, F	Shikimates and Phenylpropanoids	Flavonoids	Flavan-3-ols	UHPLC-MS/MS + RS, HR-UHPLC-MS/MS, HPLC–DAD + RS	Giordano et al. (2019), Viktorová et al. (2020), Peña-Rojas et al. (2021), Valdenegro et al. (2021)
6	Epicatechin	L	Shikimates and Phenylpropanoids	Flavonoids	Flavan-3-ols	LC-DAD + LC-MS + MS/MS + RS, UHPLC-MS/MS + RS, HR-UHPLC-MS/MS, HPLC–DAD + RS	Simirgiotis (2013), Giordano et al. (2019), Viktorová et al. (2020), Peña-Rojas et al. (2021), Valdenegro et al. (2021)
7	Procyanidin B1	L, F	Shikimates and Phenylpropanoids	Flavonoids	Proanthocyanins	LC-DAD + LC-MS + MS/MS + RS, UHPLC-MS/MS + RS, HR-UHPLC-MS/MS, HR-UHPLC-MS/MS	Simirgiotis (2013), Giordano et al. (2019), Viktorová et al. (2020), Valdenegro et al. (2021)
8	Procyanidin B2	L, F	Shikimates and Phenylpropanoids	Flavonoids	Proanthocyanins	UHPLC-MS + RS, HR-UHPLC-MS/MS, HR-UHPLC-MS/MS	Giordano et al. (2019), Viktorová et al. (2020), Valdenegro et al. (2021)
9	Procyanidin C1	L, F	Shikimates and Phenylpropanoids	Flavonoids	Proanthocyanins	UHPLC-MS/MS + RS, HR-UHPLC-MS/MS	Giordano et al. (2019), Valdenegro et al. (2021)
10	Gallic acid	L	Shikimates and Phenylpropanoids	Phenylpropanoids (C6-C1)		UHPLC-MS/MS + RS	Giordano et al. (2019)
11	Protocatechuic acid	L	Shikimates and Phenylpropanoids	Phenolic acids (C6-C1)	Simple phenolic acids	HPLC–DAD + RS	Peña-Rojas et al. (2021)
12	Caffeic acid	L	Shikimates and Phenylpropanoids	Phenylpropanoids (C6-C3)	Cinnamic acids and derivatives	UHPLC-MS/MS + RS, HPLC–DAD + RS	Giordano et al. (2019), Peña-Rojas et al. (2021)
13	*p*-coumaric acid	L	Shikimates and Phenylpropanoids	Phenylpropanoids (C6-C3)	Cinnamic acids and derivatives	HPLC–DAD + RS	Carmona et al. (2017)
14	Ferulic acid	L	Shikimates and Phenylpropanoids	Phenylpropanoids (C6-C3)	Cinnamic acids and derivatives	HPLC–DAD + RS	Peña-Rojas et al. (2021)
15	4-caffeoylquinic acid	F	Shikimates and Phenylpropanoids	Phenylpropanoids (C6-C3)	Cinnamic acids and derivatives	HR-UHPLC-MS/MS	Viktorová et al. (2020), Valdenegro et al. (2021)
16	*Trans*-chlorogenic acid	L + S, L, F	Shikimates and Phenylpropanoids	Phenylpropanoids (C6-C3)	Cinnamic acids and derivatives	^1^H and^13^C NMR + EIMS, LC-DAD + LC-MS + MS/MS + RS, UHPLC-MS/MS + RS, HPLC–DAD + RS, HR-UHPLC-MS/MS	Timmermann et al. (1995), Simirgiotis (2013), Carmona et al. (2017), Giordano et al. (2019), Viktorová et al. (2020), Peña-Rojas et al. (2021), Valdenegro et al. (2021)
17	Cryptochlorogenic acid	L	Shikimates and Phenylpropanoids	Phenylpropanoids (C6-C3)	Cinnamic acids and derivatives	UHPLC-MS/MS + RS	Giordano et al. (2019)
18	Methyl 3-caffeoylquinate | Neochlorogenic acid methyl ester	F	Shikimates and Phenylpropanoids	Phenylpropanoids (C6-C3)	Phenylpropanoids (C6-C3)	LC-DAD + LC-MS + MS/MS + RS	Simirgiotis (2013)
19	Methyl 5-caffeoylquinate | Chlorogenic acid, methyl ester	F	Shikimates and Phenylpropanoids	Phenylpropanoids (C6-C3)	Cinnamic acids and derivatives	LC-DAD + LC-MS + MS/MS + RS	Simirgiotis (2013)
20	Astilbin	L	Shikimates and Phenylpropanoids	Flavonoids	Dihydroflavonols	UHPLC-MS/MS + RS	Giordano et al. (2019)
21	Taxifolin	L	Shikimates and Phenylpropanoids	Flavonoids	Flavonoids	UHPLC-MS/MS + RS	Giordano et al. (2019)
22	Quercetin-3-*O*-galactoside | hyperoside	L + S, F	Shikimates and Phenylpropanoids	Flavonoids	Flavonols	^1^H and^13^C NMR, LC-DAD + LC-MS + MS/MS + RS, HR-UHPLC-MS/MS	Timmermann et al. (1995), Simirgiotis (2013), Viktorová et al. (2020), Valdenegro et al. (2021)
23	Isorhamnetin	L + S	Shikimates and Phenylpropanoids	Flavonoids	Flavonols	^1^H NMR	Timmermann et al. (1995)
24	Isorhamnetin-3-*O*-galactoside	L + S	Shikimates and Phenylpropanoids	Flavonoids	Flavonols	^1^H and^13^C NMR + FABMS	Timmermann et al. (1995)
25	Isorhamnetin-3-*O*-glucoside	L + S	Shikimates and Phenylpropanoids	Flavonoids	Flavonols	^1^H and^13^C NMR + FABMS	Timmermann et al. (1995)
26	Isorhamnetin-3-*O*-rhamnoside	L + S, F	Shikimates and Phenylpropanoids	Flavonoids	Flavonols	^1^H and^13^C NMR + EIMS + FABMS, HR-UHPLC-MS/MS	Timmermann et al. (1995), Viktorová et al. (2020), Valdenegro et al. (2021)
27	Isoquercitrin	L + S, F, L	Shikimates and Phenylpropanoids	Flavonoids	Flavonols	^1^H NMR, LC-DAD + LC-MS + MS/MS + RS	Timmermann et al. (1995), Simirgiotis (2013)
28	Kaempferol	L + S, L	Shikimates and Phenylpropanoids	Flavonoids	Flavonols	^1^H NMR, HPLC–DAD + RS	Timmermann et al. (1995), Carmona et al. (2017)
29	Kaempferol-3-*O*-galactoside | trifolin	L + S	Shikimates and Phenylpropanoids	Flavonoids	Flavonols	^1^H and 13C NMR + EIMS	Timmermann et al. (1995)
30	Kaempferol 3-*O*-glucoside | astragalin	F, L	Shikimates and Phenylpropanoids	Flavonoids	Flavonols	LC-DAD + LC-MS + MS/MS	Simirgiotis (2013)
31	Kaempferol-3-*O*-pentoside	L	Shikimates and Phenylpropanoids	Flavonoids	Flavonols	LC-DAD + LC-MS + MS/MS + RS	Simirgiotis (2013)
32	8-methoxykaempferol | Sexangularetin	F, L	Shikimates and Phenylpropanoids	Flavonoids	Flavonoids	LC-DAD + LC-MS + MS/MS, HR-UHPLC-MS/MS	Simirgiotis (2013), Valdenegro et al. (2021)
33	8-methoxykaempferol-3-*O*-glucoside	L	Shikimates and Phenylpropanoids	Flavonoids	Flavonols	LC-DAD + LC-MS + MS/MS	Simirgiotis (2013)
34	Myricetin	L	Shikimates and Phenylpropanoids	Flavonoids	Flavonols	LC-DAD + LC-MS + MS/MS + RS, HPLC–DAD + RS	Simirgiotis (2013), Carmona et al. (2017)
35	Luteolin 8-*C*-glucoside | orientin	F, L	Shikimates and Phenylpropanoids	Flavonoids	Flavonoids	LC-DAD + LC-MS + MS/MS + RS, HR-UHPLC-MS/MS	Simirgiotis (2013), Viktorová et al. (2020)
36	Quercetin	L + S, L	Shikimates and Phenylpropanoids	Flavonoids	Flavonols	^1^H NMR, UHPLC-MS + RS	Timmermann et al. (1995), Giordano et al. (2019)
37	Quercetin-3-*O*-pentoside | Reinutrin	F, L	Shikimates and Phenylpropanoids	Flavonoids	Flavonols	LC-DAD + LC-MS + MS/MS	Simirgiotis (2013)
38	quercetin-3-*O*-α-*D*-rhamnopyranoside | quercitrin	L + S, L	Shikimates and Phenylpropanoids	Flavonoids	Flavonoids	^1^H and^13^C NMR, UHPLC-MSMS/+RS, HPLC–DAD + RS	Timmermann et al. (1995), Carmona et al. (2017), Giordano et al. (2019), Valdenegro et al. (2021)
39	(6-(5,7-dihydroxy-2-(4-hydroxy-3-methoxyphenyl)-4-oxo-4*H*-chromen-8-yl)-3,4,5-trihydroxytetrahydro-2*H*-pyran-2-yl)methyl acetate | Isorhamnetin 3-(6″-acetylglucoside)	F	Shikimates and Phenylpropanoids	Flavonoids	Flavonols	HR-UHPLC-MS/MS	Valdenegro et al. (2021)
40	Rutin	L	Shikimates and Phenylpropanoids	Flavonoids	Flavonols	UHPLC-MS/MS + RS, HPLC–DAD + RS	Carmona et al. (2017), Giordano et al. (2019)
41	Apigenin 8-*C*-glucoside | vitexin	L	Shikimates and Phenylpropanoids	Flavonoids	Flavones	LC-DAD + LC-MS + MS/MS	Simirgiotis (2013)
42	Cryptofolione	F	Shikimates and Phenylpropanoids	Styrylpyrones	Kavalactones and derivatives	^1^H and^13^C NMR + MS	Schmeda-Hirschmann et al. (2001)
43	6-(4,6-dimethoxy-8-phenyl-octa-1,7-dienyl)-4-hydroxy-tetrahydro-pyran-2-one	F	Shikimates and Phenylpropanoids	-	-	^1^H and^13^C NMR + MS	Schmeda-Hirschmann et al. (2001)
44	cryptorigidifoliol A	F	Polyketides	Cyclic polyketides	2-pyrone derivatives	HR-UHPLC-MS/MS	Valdenegro et al. (2021)
45	(4*R*,6*S*)-10-phenyl-1-decene-4,6-diol	F	Polyketides	-	-	HR-UHPLC-MS/MS	Valdenegro et al. (2021)
46	ethyl 5-hydroxy-7-phenyl-2,6-heptadienoate	F	Shikimates and Phenylpropanoids	Phenylpropanoids (C6-C3)	Cinnamic acids and derivatives	HR-UHPLC-MS/MS	Valdenegro et al. (2021)
47	1′R*,3′S*,4′R*,5′S*,6*S*-6-[(4′-ethyl-9′-oxabicycle [3.3.1]non-6′-en-3′-yl)methyl]- 5,6-dihydro-2*H*-pyran-2-one	F	Polyketides	Macrolides	Macrolide lactones	HR-UHPLC-MS/MS	Valdenegro et al. (2021)
48	(+)-lariciresinol	F	Shikimates and Phenylpropanoids	Lignans	Furanoid lignans	HR-UHPLC-MS/MS	Viktorová et al. (2020), Valdenegro et al. (2021)
49	(−)-rubrichalcolactone	F	Shikimates and Phenylpropanoids	Flavonoids	Chalcones	HR-UHPLC-MS/MS	Valdenegro et al. (2021)

B: bark, F: fruits, L: leaves, R: roots, W: wood.

#### 3.1.2 Alkaloids in the aerial parts of *Cryptocarya alba*

Alkaloids constitute a large group of secondary metabolites found in various plant species and contribute to their chemical and medicinal properties, which are highly valued by the scientific community (Majnooni et al., 2021). In particular, benzylisoquinoline alkaloids are a diverse class of secondary metabolites with a broad spectrum of specialized biological activities (Quiroz-Carreño et al., 2020). These isoquinoline alkaloids are frequently identified within the Lauraceae family, primarily within the groups of benzyl-tetrahydroisoquinolines, aporphines, and pavines (Villamizar, 2010; Custodio and da Veiga Junior, 2014; van der Merwe et al., 2016; Cassels et al., 2021; da Silva Antonio et al., 2024). Among these species, *C. alba* and *P. boldus* are notable, as they are two endemic native species with unique features, belonging to the sclerophyllous forests of central Chile (Urzúa et al., 1974). These species share their habitat across the country and display similar chemical profiles, consisting of a significant number of alkaloids of the aporphine and tetrahydroisoquinoline types (Cassels et al., 2019; Quiroz-Carreño et al., 2020). However, the alkaloid levels in *C. alba* are considerably lower, necessitating much more effort for their isolation and purification (Castro-Saavedra et al., 2016b; Fuentes-Barros et al., 2018; Giordano et al., 2019).

Examples of *Cryptocarya* species containing pavine-type alkaloids include neocaryachine, which has been isolated from *Cryptocarya chinensis* (Hance) Hemsl. (Lee et al., 1990), *Cryptocarya laevigata* Blume (Suzuki et al., 2017), and *Cryptocarya wrayi* Gamble (Liu et al., 2022). Aporphinic alkaloids have been identified in *Cryptocarya moschata* Nees & Mart. and *Cryptocarya mandioccana* Meisn. (Zoccolotti et al., 2021), *Cryptocarya bracteolata* Gamble (Saidi et al., 2016), *C. diversifolia* Blume (syn. *Cryptocarya crassinervia* Miq.) (Awang et al., 2008), *Cryptocarya ferrea* Blume (Saidi et al., 2009; 2019), *Cryptocarya densiflora* Blume (Othman et al., 2017), *C. chinensis* (Lin et al., 2001), *Cryptocarya longifolia* Kosterm. (Bick et al., 1981), *C. triplinervis* R.Br. (Cooke and Haynes, 1954), and *Cryptocarya angulata* C.T.White (Cooke and Haynes, 1954). Reticuline, laurotetanine, and *N*-methyllaurotetanine—considered important for the genus *Cryptocarya* and similar to *C. alba*—were detected in the ground-dried bark of *Cryptocarya griffithiana* Wight (Othman et al., 2023). Additionally, an alkaloid called isocryprochine has been isolated from *C. chinensis*, an evergreen tree widely found in lowland forests of Taiwan and southern China, and used in traditional Taiwanese medicine (Wu and Lin, 2001). The alkaloid cryprochine has also been extracted from the leaves and bark of *C. chinensis* (Lee et al., 1993).

Despite the clear interest and potential of *C. alba*, this traditional species had been overlooked in chemical studies of its components for many years (Urzúa et al., 1974). The scientific literature contains only one reference reporting the detection of tannins and resins in the leaves, bark, and fruits of this species, published in 1956 (Gautier and Pardo, 1956). This early chemical analysis, aimed at identifying the active compounds responsible for the fruit’s antirheumatic effects, reported the presence of resins (2.96%), fatty substances (17.63%), tannins, and an uncharacterized glycoside (Gautier and Pardo, 1956). Initial efforts in phytochemical research on alkaloids in native Chilean Lauraceae species, specifically *C. alba*, began with the collection of 1.3 kg of trunk bark in 1972 from the El Toro stream in Cajón del Maipo, Region Metropolitana, Chile. After extensive laboratory work, only one basic compound was identified, isolated, and characterized as (+)-reticuline (Urzúa et al., 1974). Reticuline is a benzylisoquinoline alkaloid, an amorphous powder first isolated in small amounts from 7 kg of dried bark of *Annona reticulata* L. [Annonaceae] (Gopinath et al., 1959). A few years later, it was also isolated from other species such as the opium poppy (*Papaver somniferum* L. [Papaveraceae]) (Brochmann-Hanssen and Nielsen, 1965) and from the leaves of *P. boldus* (Hughes et al., 1968). During that period, reticuline was recognized as a common precursor in the biosynthesis pathways of multiple alkaloids, including morphine (Kirby, 1967). Years later, 0.003%, 0.025%, 0.009%, and 0.057% of crude extract of alkaloids were isolated from the leaves, roots, wood, and bark of *C. alba*, respectively (Castro-Saavedra et al., 2016b), which falls within the established range for alkaloidal species (>0.001%) (Majnooni et al., 2021).

**FIGURE 5:**
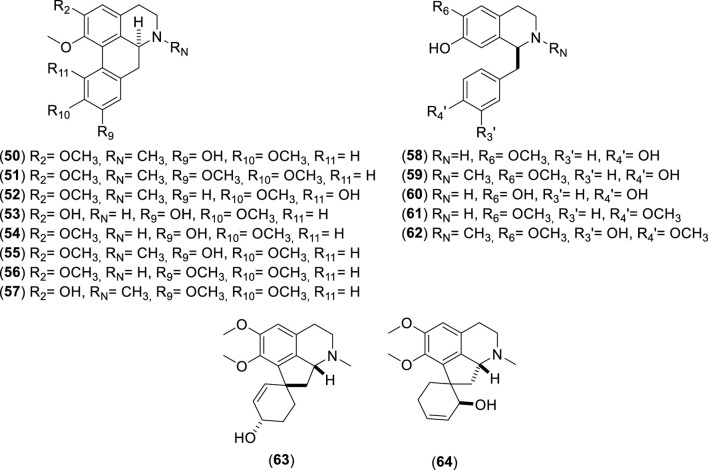
Chemical structures of alkaloids (50–64) identified from *Cryptocarya alba*.

Analyzing pure alkaloid standards by ultra-high performance liquid chromatography–tandem mass spectrometry (UHPLC/MS-MS) analysis, twelve alkaloids were identified: four benzyl-tetrahydroisoquinolines—reticuline, coclaurine, *N*-methylcoclaurine, and norreticuline—and eight aporphines—boldine, isocorydine, laurolitsine, laurotetanine, *N*-methyllaurotetanine, predicentrine, norglaucine, and glaucine (Castro-Saavedra et al., 2016b). The leaves of young trees contain higher concentrations of laurolitsine and laurotetanine. Laurolitsine, along with reticuline, is the most abundant alkaloid in the wood. Reticuline, laurotetanine, *N*-methyllaurotetanine, and norglaucine are the main alkaloids in the bark of trees with a diameter of less than 10 cm. While reticuline, laurotetanine, boldine, and *N*-methyllaurotetanine are the dominant alkaloids in the bark of long-lived adult trees (Castro-Saavedra et al., 2016b), significant variations between different parts of the plant and between individuals were observed. Concentrations of alkaloids change throughout the tree’s life; some, such as boldine, predicentrine, laurotetanine, and reticuline, tend to accumulate significantly in the bark of older trees. At this stage, some trees contain small amounts of glaucine (Castro-Saavedra et al., 2016b). The highest concentrations in the bark of the oldest individuals were observed, with reticuline reaching 542 μg/g dry material (Castro-Saavedra et al., 2016b). Once the profile was identified, population level studies were conducted to verify the results on a larger scale and better characterize the species. The alkaloid profile in branches (wood and bark) was consistent with previous studies, with reticuline as the main alkaloid of *C. alba* (Giordano et al., 2019). The alkaloids present in *C. alba* are shown in Table 2, and structures are provided in Figure 5.

**TABLE 2:** Alkaloids reported in *Cryptocarya alba*.

n°	Compound	Par	Alkaloid class	Identification	References
50	Boldine	B, L, R, W	Aporphine	^1^H and^13^C NMR + UHPLC-MS/MS + RS, UHPLC-MS/MS + RS	Castro-Saavedra et al. (2016b), Giordano et al. (2019)
51	Glaucine	B, L, R, W	Aporphine	^1^H and^13^C NMR + UHPLC-MS/MS + RS	Castro-Saavedra et al. (2016b)
52	Isocorydine	B, L, R, W	Aporphine	^1^H and^13^C NMR + UHPLC-MS/MS + RS, UHPLC-MS/MS + RS	Castro-Saavedra et al. (2016b), Giordano et al. (2019)
53	Laurolitsine	B, L, W	Aporphine	^1^H and^13^C NMR + UHPLC-MS/MS + RS, UHPLC-MS/MS + RS	Castro-Saavedra et al. (2016b), Giordano et al. (2019)
54	Laurotetanine	B, L, W	Aporphine	^1^H and^13^C NMR + UHPLC-MS + RS, UHPLC-MS + RS	Castro-Saavedra et al. (2016b), Giordano et al. (2019)
55	*N*-methyllaurotetanine	B, L, R, W	Aporphine	^1^H and^13^C NMR + UHPLC-MS/MS + RS, UHPLC-MS/MS + RS	Castro-Saavedra et al. (2016b), Giordano et al. (2019)
56	Norglaucine	B, L, R, W	Aporphine	^1^H and^13^C NMR + UHPLC-MS/MS + RS	Castro-Saavedra et al. (2016b)
57	Predicentrine	B, R, W	Aporphine	^1^H and^13^C NMR + UHPLC-MS/MS + RS	Castro-Saavedra et al. (2016b)
58	Coclaurine	B, L, R, W	Tetrahydroisoquinoline	^1^H and^13^C NMR + UHPLC-MS/MS + RS, UHPLC-MS/MS + RS	Castro-Saavedra et al. (2016b), Giordano et al. (2019)
59	*N*-methylcoclaurine	B, R, W	Tetrahydroisoquinoline	^1^H and^13^C NMR + UHPLC-MS/MS + RS, UHPLC-MS/MS + RS	Castro-Saavedra et al. (2016b), Giordano et al. (2019)
60	Norcoclaurine (higenamine)	L	Tetrahydroisoquinoline	UHPLC-MS/MS + RS	Giordano et al. (2019)
61	Norreticuline	R	Tetrahydroisoquinoline	^1^H and^13^C NMR + UHPLC-MS/MS + RS	Castro-Saavedra et al. (2016b)
62	Reticuline	B, L, R, W	Tetrahydroisoquinoline	^1^H NMR, ^1^H and^13^C NMR + UHPLC-MS/MS + RS, UHPLC-MS/MS + RS	Urzúa et al. (1974), Castro-Saavedra et al. (2016b), Giordano et al. (2019)
63	Cryprochine	F	Isoquinoline	HR-UHPLC-MS/MS	Valdenegro et al. (2021)
64	Isocryprochine	F	Isoquinoline	HR-UHPLC-MS/MS	Valdenegro et al. (2021)

B: bark, F: fruits, L: leaves, R: roots, W: wood.

#### 3.1.3 Essential oils in the aerial parts of *Cryptocarya alba*

In the case of *C. alba* EOs, variations of over 100% have been observed depending on collection time. The lowest yield was recorded in August at 1.6 mL%, while the highest was in July at 3.9 mL% (Montes et al., 1988). Eight studies have analyzed EOs from leaves, but to date, no study has examined EOs from the bark.

**FIGURE 6:**
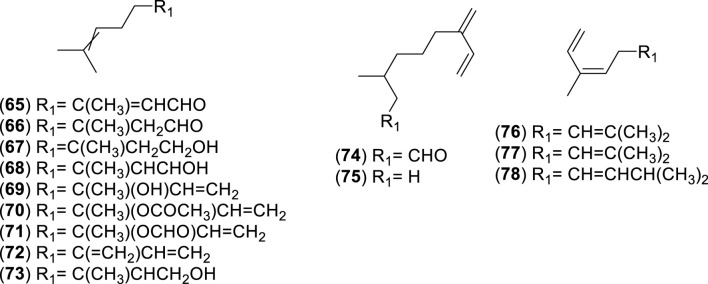
Chemical structures of acyclic monoterpenes (AMs, 65–78) identified in the essential oils of *Cryptocarya alba*.

**FIGURE 7:**
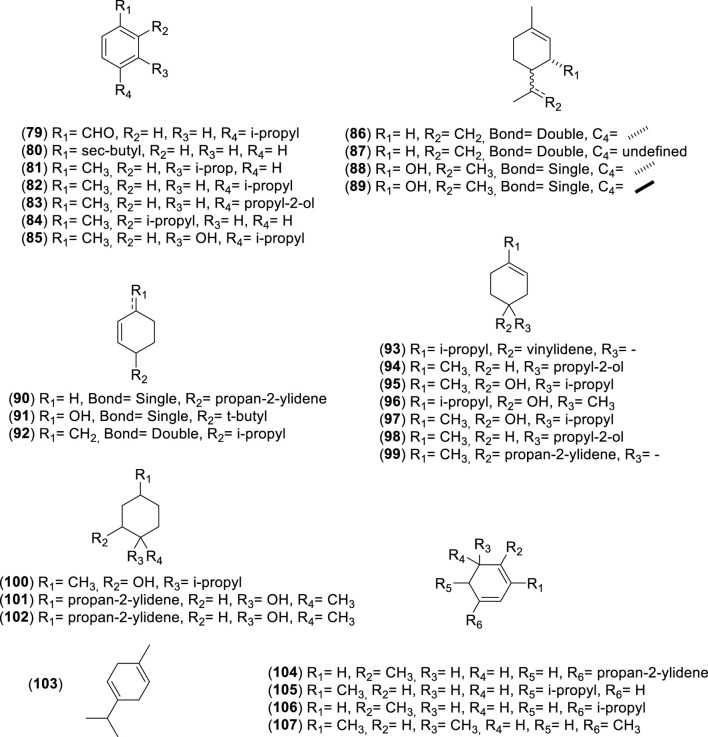
Chemical structures of monocyclic monoterpenes (MMs, 79–107) identified in the essential oils of *Cryptocarya alba*.

**FIGURE 8:**
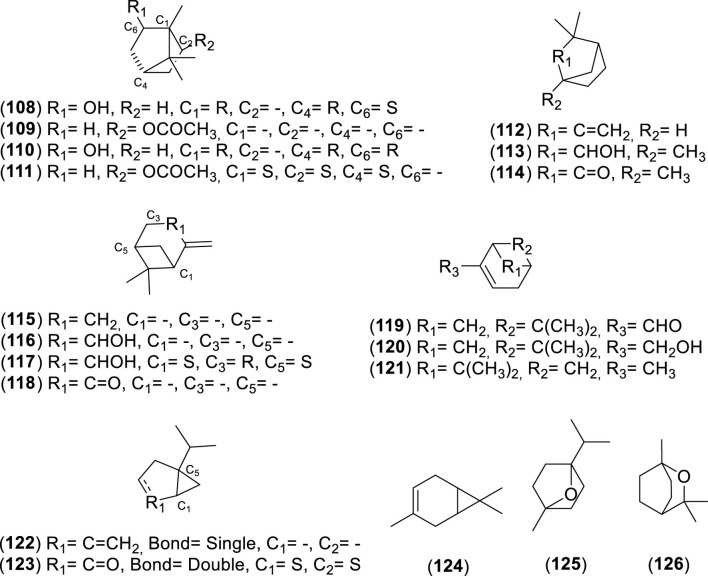
Chemical structures of bicyclic monoterpenes (BMs, 108–126) identified in the essential oils of *Cryptocarya alba*.

**FIGURE 9:**
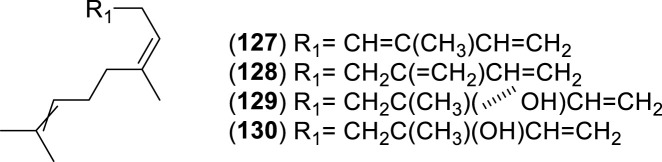
Chemical structures of acyclic sesquiterpenes (ASs, 127–130) identified in the essential oils of *Cryptocarya alba*.

**FIGURE 10:**
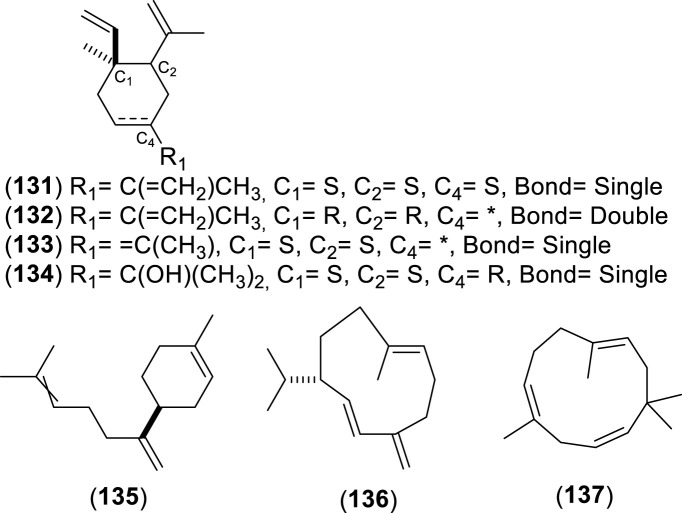
Chemical structures of monocyclic sesquiterpenes (MSs, 131–134) identified in the essential oils of *Cryptocarya alb*a.

**FIGURE 11:**
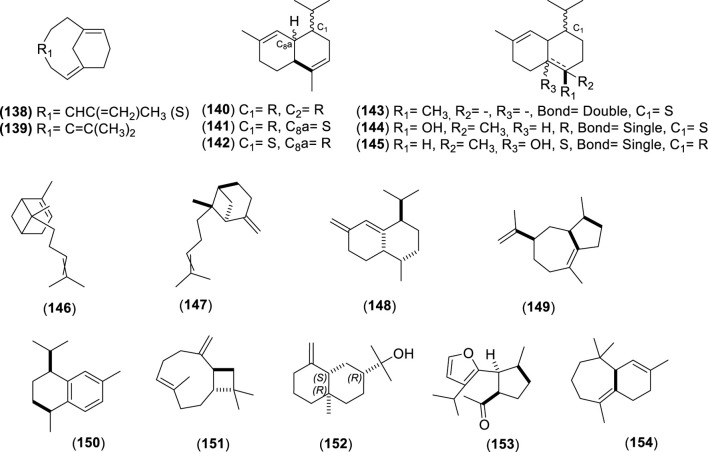
Chemical structures of bicyclic sesquiterpenes (BSs, 138–154) identified in the essential oils of *Cryptocarya alba*.

**FIGURE 12:**
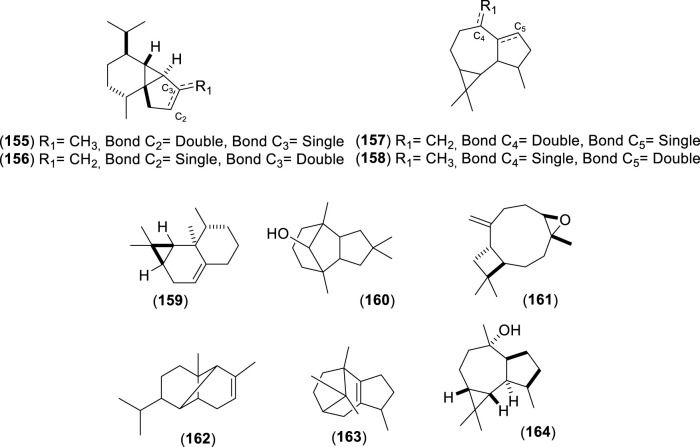
Chemical structures of tricyclic sesquiterpenes (TSs, 159–164) identified in the essential oils of *Cryptocarya alba*.

**FIGURE 13:**
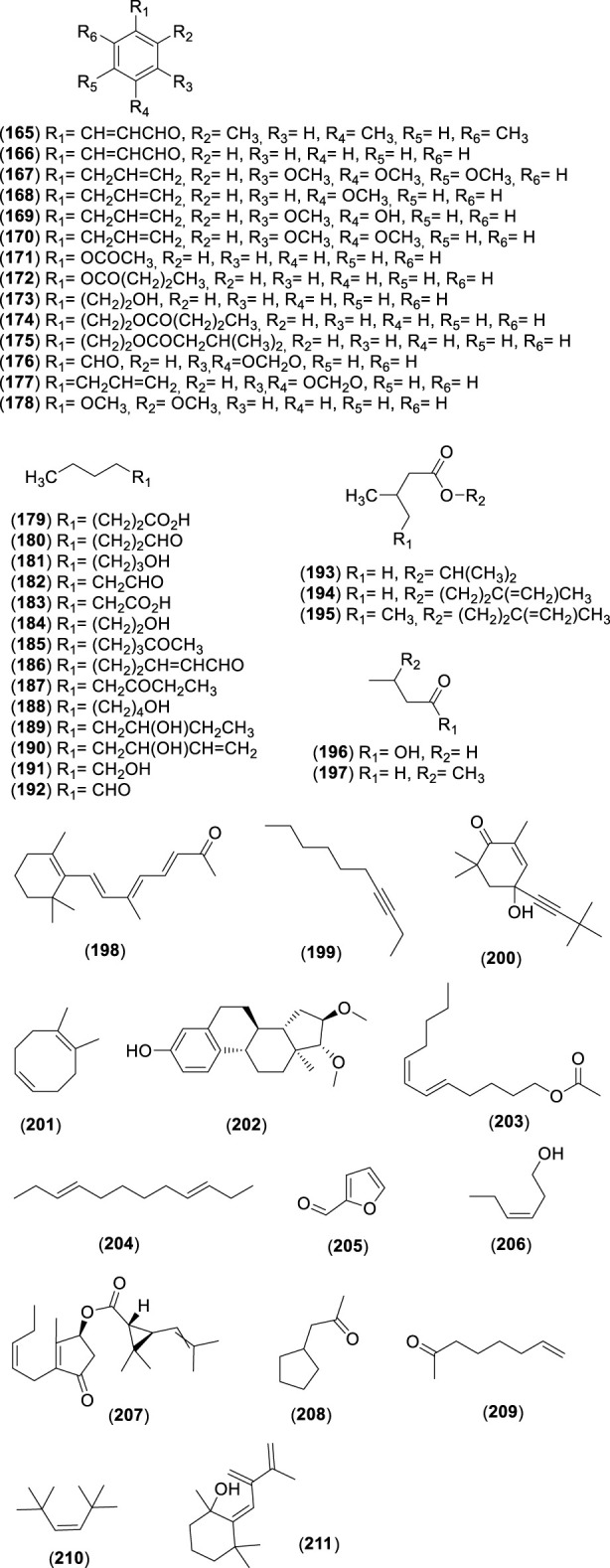
Chemical structures of miscellaneous compounds (MCs, 165–211) identified in the essential oils of *Cryptocarya alba*.

The first study by Montes et al. (1988) reported an average EO yield of 0.26% across seven samples. Using gas chromatography-mass spectrometry (GC-MS) and column chromatography, seventy-one components were identified. Of these, 25.35% were terpene hydrocarbons, 33.8% alcohols, 18.3% carbonyl compounds, 8.45% esters, 4.2% acids, 5.6% phenols, 1.4% oxides, and 2.8% ethers. The main component in these EO samples was 1-terpinen-4-ol (14.1%), followed by *p*-cymene (7.3%), cineole (6.6%), α-pinene (3.8%), β-pinene (2.1%), and borneol-terpineol (3.01%). An EO from leaves of *C. alba* collected in Olmué, Región de Valparaíso, Chile, yielded 0.4%, with the primary components being 1,8-cineole (21.4%), 4-terpineol (18.2%), beta-pinene (17.5%), and alpha-pinene (8.2%) (Niemeyer and Teillier, 2007). Another EO from *C. alba* leaves collected in Nonguén Valley, Región del BíoBío, Chile, had a yield of 0.76%. It was mainly composed of monoterpenoids such as 1-terpinen-4-ol (28.2%), beta-terpinene (23.1%), eucalyptol (18.9%), *p*-cymene (16.0%), and alpha-pinene (11.1%) (Avello Lorca et al., 2012). In Cuesta Lo Prado, Región Metropolitana, Chile, leaves yielded 0.17% EO, with 38 identified compounds differing from those reported previously. Notable components included 4-terpineol (17.5%), 4-(3,3-dimethyl-but-1-ynyl)-4-hydroxy-2,6,6-trimethylcyclohex-2-enone (12.8%), 1,8-cineole (7.9%), *p*-cymene (7.1%), and sabinene (6.8%), collectively making up 52.1% of the EO (Di Cosmo et al., 2015). In Pinto, Región del Ñuble, Chile, although yield was not reported, the main constituents included (*E*)-beta-bergamotene (15.6%), viridiflorol (8.5%), germacrene-D (7.7%), beta-apo-13-carotenone (5.3%), linalool (4.4%), (−)-terpinen-4-ol (3.5%), 2-methyl-cyclopentane propanone (3.4%), alpha-farnesene (2.9%), beta-himachelene (2.7%), 1,8-cineole (1.9%), beta-cubebene (1.5%), jasmolin (1.5%), and safrole (1.1%) (Pinto et al., 2016). A study of EO from leaves collected in Altos de Chicauma, Región Metropolitana, Chile, reported no yield but identified 39 compounds. The EO was rich in alpha-terpineol (27.4%), eucalyptol (23.3%), and beta-phellandrene (16.3%) (Bravo et al., 2017). From eight leaf samples collected across three sectors of the Región Metropolitana, sixteen compounds were identified in EOs of at least four samples with a relative concentration of 1% or more. In six samples, sabinene was the primary or secondary component, averaging 13.5% ± 2.8%. In the remaining two, camphene was most abundant (20.6% and 22.7%), followed by beta-eudesmol (6.1% ± 5.7%) and eucalyptol (5.4% ± 1.5%) (Giordano et al., 2019). Lastly, the EO from leaves collected in Altos de Chicauma had a yield of 0.6%, with 14 main compounds, primarily alpha-terpineol (25.0%), eucalyptol (21.6%), and beta-phellandrene (14.8%) (Touma et al., 2020). Monoterpenes, a type of biogenic volatile organic compounds, contribute to ozone formation. In *C. alba*, the emission of these monoterpenes varies with tree age and season (Préndez et al., 2013). The compounds present in the EOs of *C. alba* are detailed in Tables 3–5, and structures of monoterpenoids (Figures 6–8), sesquiterpenoids (Figures 9–12), and miscellaneous compounds (Figure 13) are provided.

**TABLE 3:** Monoterpenes identified in the essential oils from *Cryptocarya alba*.

n°	Compound	Pathway	Superclass	Class	Identification	References
65	Citral	Terpenoids	Monoterpenoids	Acyclic	GC-MS	Montes et al. (1988)
66	Citronellal	Terpenoids	Monoterpenoids	Acyclic	GC-MS	Montes et al. (1988)
67	Citronellol	Terpenoids	Monoterpenoids	Acyclic	GC-MS	Montes et al. (1988)
68	Geraniol	Terpenoids	Monoterpenoids	Acyclic	GC-MS	Montes et al. (1988)
69	Linalool	Terpenoids	Monoterpenoids	Acyclic	GC-MS + RI, GC-MS + RI + Co-I	Montes et al. (1988), Niemeyer and Teillier (2007), Di Cosmo et al. (2015), Pinto et al. (2016)
70	Linalyl acetate	Terpenoids	Monoterpenoids	Acyclic	GC-MS	Montes et al. (1988)
71	Linalyl formate	Terpenoids	Monoterpenoids	Acyclic	GC-MS + RI + Co-I	Pinto et al. (2016)
72	β-myrcene	Terpenoids	Monoterpenoids	Acyclic	GC-MS + RI	Niemeyer and Teillier (2007), Di Cosmo et al. (2015)
73	Nerol	Terpenoids	Monoterpenoids	Acyclic	GC-MS	Montes et al. (1988)
74	Myrcenal	Fatty acids	Fatty acyls	Fatty aldehydes	GC-MS + RI	Di Cosmo et al. (2015)
75	Myrcene	Terpenoids	Monoterpenoids	Acyclic	GC-MS	Montes et al. (1988)
76	(*E*)-ocimene	Terpenoids	Monoterpenoids	Acyclic	GC-MS + RI	Niemeyer and Teillier (2007)
77	(*Z*)-ocimene	Terpenoids	Monoterpenoids	Acyclic	GC-MS + RI	Niemeyer and Teillier (2007)
78	Ocimene	Terpenoids	Monoterpenoids	Acyclic	GC-MS	Montes et al. (1988)
79	Cuminaldehyde	Terpenoids	Monoterpenoids	Menthane	GC-MS	Montes et al. (1988)
80	α-cymene	Shikimates and Phenylpropanoids	Miscellaneous	-	GC-MS + RI	Niemeyer and Teillier (2007)
81	β-cymene	Terpenoids	Monoterpenoids	Menthane	GC-MS + RI	Giordano et al. (2019)
82	*P*-cymene	Terpenoids	Monoterpenoids	Menthane	GC-MS, GC-MS + RI + Co-I, GC-MS + RI	Montes et al. (1988), Avello Lorca et al. (2012), Di Cosmo et al. (2015)
83	*P*-cymen-8-ol	Terpenoids	Sesquiterpenoids	Bisabolane	GC-MS + RI	Di Cosmo et al. (2015)
84	*o*-cymol	Terpenoids	Monoterpenoids	Menthane	GC-MS + RI	Bravo et al. (2017), Touma et al. (2020)
85	Thymol	Terpenoids	Monoterpenoids	Menthane monoterpenoids	GC-MS	Montes et al. (1988)
86	(+)-limonene	Terpenoids	Monoterpenoids	Menthane and monocyclic	GC-MS, GC-MS + RI	Montes et al. (1988), Bravo et al. (2017), Touma et al. (2020)
87	(−)-*DL*-limonene	Terpenoids	Monoterpenoids	Menthane and monocyclic	GC-MS	Montes et al. (1988)
88	*cis*-piperitol	Terpenoids	Monoterpenoids	Menthane monoterpenoids	GC-MS	Montes et al. (1988)
89	*Trans*-piperitol	Terpenoids	Monoterpenoids	Menthane monoterpenoids	GC-MS + RI	Di Cosmo et al. (2015)
90	Isoterpinolene	Terpenoids	Monoterpenoids	Menthane	GC-MS + RI + Co-I	Pinto et al. (2016)
91	*cis*-*p*-2-menthen-1-ol	Terpenoids	Miscellaneous	-	GC-MS + RI + Co-I	Pinto et al. (2016)
92	β-phellandrene	Terpenoids	Monoterpenoids	Monocyclic monoterpenoids	GC-MS + RI	Niemeyer and Teillier (2007), Pinto et al. (2016), Bravo et al. (2017), Touma et al. (2020)
93	β-terpinene	Terpenoids	Monoterpenoids	Monocyclic monoterpenoids	GC-MS + RI + Co-I	Avello Lorca et al. (2012), Pinto et al. (2016)
94	α-terpineol	Terpenoids	Monoterpenoids	Methane monoterpenoids	GC-MS + RI	Touma et al. (2020)
95	(−)-terpinen-4-ol	Terpenoids	Monoterpenoids	Methane monoterpenoids	GC-MS + RI + Co-I	Pinto et al. (2016)
96	1-terpineol	Terpenoids	Monoterpenoids	Methane monoterpenoids	GC-MS + RI + Co-I	Di Cosmo et al. (2015)
97	4-terpineol	Terpenoids	Monoterpenoids	Methane monoterpenoids	GC-MS, GC-MS + RI + Co-I + NMR	Montes et al. (1988), Niemeyer and Teillier (2007), Avello Lorca et al. (2012), Di Cosmo et al. (2015), Bravo et al. (2017), Giordano et al. (2019), Touma et al. (2020)
98	α-terpineol	Terpenoids	Monoterpenoids	Methane monoterpenoids	GC-MS + RI + Co-I	Montes et al. (1988), Niemeyer and Teillier (2007), Di Cosmo et al. (2015), Pinto et al. (2016), Bravo et al. (2017)
99	terpinolene	Terpenoids	Monoterpenoids	-	GC-MS, GC-MS + RI	Montes et al. (1988), Niemeyer and Teillier (2007), Bravo et al. (2017), Touma et al. (2020)
100	Menthol	Terpenoids	Monoterpenoids	Menthane and monocyclic	GC-MS	Montes et al. (1988)
101	β-terpineol	Terpenoids	Monoterpenoids	Methane monoterpenoids	GC-MS + RI	Di Cosmo et al. (2015)
102	*cis*-β-terpineol	Terpenoids	Monoterpenoids	Methane monoterpenoids	GC-MS + RI + Co-I	Di Cosmo et al. (2015)
103	γ-terpinene	Terpenoids	Monoterpenoids	Monocyclic monoterpenoids	GC-MS + RI	Montes et al. (1988), Niemeyer and Teillier (2007), Bravo et al. (2017), Giordano et al. (2019), Touma et al. (2020)
104	1,3,8-*p*-menthatriene	Terpenoids	Monoterpenoids	-	GC-MS + RI + Co-I	Pinto et al. (2016)
105	α-phellandrene	Terpenoids	Monoterpenoids	Monocyclic monoterpenoids	GC-MS, GC-MS + RI	Montes et al. (1988), Niemeyer and Teillier (2007), Pinto et al. (2016), Bravo et al. (2017)
106	α-terpinene	Terpenoids	Monoterpenoids	Monocyclic monoterpenoids	GC-MS + RI	Niemeyer and Teillier (2007)
107	1,3,5,5-tetramethyl-1,3-cyclohexadiene	Terpenoids	Monoterpenoids	-	GC-MS + RI	Niemeyer and Teillier (2007)
108	Borneol	Terpenoids	Monoterpenoids	Camphane	GC-MS	Montes et al. (1988)
109	Bornyl acetate	Terpenoids	Monoterpenoids	Camphane	GC-MS	Montes et al. (1988)
110	Isoborneol	Terpenoids	Monoterpenoids	Camphane	GC-MS	Montes et al. (1988)
111	Isobornyl acetate	Terpenoids	Monoterpenoids	Camphane	GC-MS	Montes et al. (1988)
112	Camphene	Terpenoids	Monoterpenoids	Camphane/Fenchane	GC-MS, GC-MS + RI	Montes et al. (1988), Niemeyer and Teillier (2007), Di Cosmo et al. (2015), Bravo et al. (2017), Touma et al. (2020)
113	Fenchyl alcohol	Terpenoids	Monoterpenoids	Fenchane	GC-MS	Montes et al. (1988)
114	Fenchone	Terpenoids	Monoterpenoids	Fenchane	GC-MS	Montes et al. (1988)
115	β-pinene	Terpenoids	Monoterpenoids	Pinane monoterpenoids	GC-MS, GC-MS + RI, GC-MS + RI + Co-I	Montes et al. (1988), Niemeyer and Teillier (2007), Avello Lorca et al. (2012), Di Cosmo et al. (2015), Bravo et al. (2017), Touma et al. (2020)
116	Pinocarveol	Terpenoids	Monoterpenoids	Pinane monoterpenoids	GC-MS, GC-MS + RI + Co-I	Montes et al. (1988), Pinto et al. (2016)
117	*Trans*-pinocarveol	Terpenoids	Monoterpenoids	Pinane monoterpenoids	GC-MS + RI	Niemeyer and Teillier (2007), Di Cosmo et al. (2015)
118	Pinocarvone	Terpenoids	Monoterpenoids	Menthane monoterpenoids	GC-MS, GC-MS + RI	Montes et al. (1988), Niemeyer and Teillier (2007)
119	Myrtenal	Terpenoids	Monoterpenoids	Pinane	GC-MS	Montes et al. (1988)
120	Myrtenol	Terpenoids	Monoterpenoids	Pinane	GC-MS	Montes et al. (1988)
121	α-pinene	Terpenoids	Monoterpenoids	Pinane monoterpenoids	GC-MS, GC-MS + RI, GC-MS + RI + Co-I	Montes et al. (1988), Niemeyer and Teillier (2007), Avello Lorca et al. (2012), Di Cosmo et al. (2015), Bravo et al. (2017), Giordano et al. (2019)
122	Sabinene	Terpenoids	Monoterpenoids	Menthane monoterpenoids	GC-MS, GC-MS + RI, GC-MS + RI + Co-I	Montes et al. (1988), Di Cosmo et al. (2015), Pinto et al. (2016), Giordano et al. (2019)
122	α-thujene	Terpenoids	Monoterpenoids	Thujane monoterpenoids	GC-MS, GC-MS + RI	Montes et al. (1988), Niemeyer and Teillier (2007), Di Cosmo et al. (2015)
124	3-carene	Terpenoids	Monoterpenoids	Carane	GC-MS + RI	Montes et al. (1988), Niemeyer and Teillier (2007)
125	1,4-cineole	Terpenoids	Monoterpenoids	Menthane	GC-MS + RI	Di Cosmo et al. (2015)
126	1,8-cineole | eucalyptol	Terpenoids	Monoterpenoids	Menthane	GC-MS + RI, GC-MS + RI + Co-I, GC-MS + RI + Co-I + NMR	Montes et al. (1988), Niemeyer and Teillier (2007), Avello Lorca et al. (2012), Di Cosmo et al. (2015), Pinto et al. (2016), Bravo et al. (2017), Giordano et al. (2019), Touma et al. (2020)

Co-I: co-injection; GC-MS: gas chromatography mass spectrometry, RI: retention index.

**TABLE 4:** Sesquiterpenes identified in the essential oils from *Cryptocarya alba*.

n°	Compound	Pathway	Superclass	Class	Identification	References
127	α-farnesene	Terpenoids	Sesquiterpenoids	Farnesane	GC-MS + RI + Co-I, GC-MS + RI	Pinto et al. (2016), Giordano et al. (2019)
128	β-farnesene	Terpenoids	Sesquiterpenoids	Farnesane	GC-MS + RI	Di Cosmo et al. (2015), Giordano et al. (2019)
129	(*Z*)-nerolidol	Terpenoids	Sesquiterpenoids	Acyclic/Farnesane	GC-MS + RI	Niemeyer and Teillier (2007)
130	Nerolidol	Terpenoids	Sesquiterpenoids	Acyclic/Farnesane	GC-MS	Montes et al. (1988)
131	β-elemene	Terpenoids	Sesquiterpenoids	Elemane	GC-MS + RI	Niemeyer and Teillier (2007), Di Cosmo et al. (2015)
132	δ-elemene	Terpenoids	Sesquiterpenoids	Elemane	GC-MS + RI + Co-I	Pinto et al. (2016)
133	γ-elemene	Terpenoids	Sesquiterpenoids	Elemane	GC-MS + RI	Bravo et al. (2017), Touma et al. (2020)
134	Elemol	Terpenoids	Sesquiterpenoids	Elemane	GC-MS	Montes et al. (1988)
135	β-bisabolene	Terpenoids	Sesquiterpenoids	Bisabolane	GC-MS + RI + Co-I	Pinto et al. (2016)
136	Germacrene D	Terpenoids	Sesquiterpenoids	Germacrene	GC-MS + RI + Co-I	Pinto et al. (2016)
137	α-humulene	Terpenoids	Sesquiterpenoids	Humulane	GC-MS + RI	Niemeyer and Teillier (2007)
138	(−)-germacrene A	Terpenoids	Sesquiterpenoids	Germacrene	GC-MS + RI + Co-I	Pinto et al. (2016)
139	Germacrene B	Terpenoids	Sesquiterpenoids	Germacrene	GC-MS + RI	Niemeyer and Teillier (2007)
140	α-amorphene	Terpenoids	Sesquiterpenoids	Cadinane	GC-MS + RI	Di Cosmo et al. (2015)
141	α-cadinene	Terpenoids	Sesquiterpenoids	Cadinane	GC-MS, GC-MS + RI + Co-I	Montes et al. (1988), Pinto et al. (2016)
142	α-muurolene	Terpenoids	Sesquiterpenoids	Cadinane	GC-MS + RI	Di Cosmo et al. (2015)
143	δ-cadinene	Terpenoids	Sesquiterpenoids	Cadinane	GC-MS + RI + Co-I	Pinto et al. (2016)
144	α-cadinol	Terpenoids	Sesquiterpenoids	Cadinane	GC-MS + RI	Di Cosmo et al. (2015)
145	Cubenol	Terpenoids	Sesquiterpenoids	Cadinane and Zizaane	GC-MS + RI	Di Cosmo et al. (2015)
146	α-bergamotene	Terpenoids	Sesquiterpenoids	Bergamotane	GC-MS + RI	Niemeyer and Teillier (2007), Di Cosmo et al. (2015), Bravo et al. (2017), Touma et al. (2020)
147	*E*-β-bergamotene	Terpenoids	Sesquiterpenoids	Bergamotane	GC-MS + RI + Co-I	Pinto et al. (2016)
148	Bicyclosesquiphellandrene	Terpenoids	Sesquiterpenoids	Cadinane	GC-MS + RI + Co-I	Pinto et al. (2016)
149	α-bulnesene	Terpenoids	Sesquiterpenoids	Guaiane	GC-MS + RI + Co-I	Pinto et al. (2016)
150	*Cis*-calamenene	Terpenoids	Sesquiterpenoids	Cadinane	GC-MS + RI	Niemeyer and Teillier (2007), Di Cosmo et al. (2015), Bravo et al. (2017), Giordano et al. (2019), Touma et al. (2020)
151	β-caryophyllene	Terpenoids	Sesquiterpenoids	Humulane	GC-MS + RI	Niemeyer and Teillier (2007), Di Cosmo et al. (2015)
152	β-eudesmol	Terpenoids	Sesquiterpenoids	Eudesmane	GC-MS + RI	Niemeyer and Teillier (2007), Bravo et al. (2017), Giordano et al. (2019), Touma et al. (2020)
153	Furopelargone A	Terpenoids	Sesquiterpenoids		GC-MS + RI	Giordano et al. (2019)
154	β-himachalene	Terpenoids	Sesquiterpenoids	Himachalene	GC-MS + RI + Co-I	Pinto et al. (2016)
155	α-cubebene	Terpenoids	Sesquiterpenoids	Cubebane	GC-MS + RI, GC-MS + RI + Co-I	Niemeyer and Teillier (2007), Di Cosmo et al. (2015), Pinto et al. (2016)
156	β-cubebene	Terpenoids	Sesquiterpenoids	Cubebane	GC-MS + RI + Co-I	Pinto et al. (2016)
157	Dehydro-aromadendrene	Terpenoids	Sesquiterpenoids	Aromadendrane	GC-MS + RI + Co-I	Pinto et al. (2016)
158	Isoledene	Terpenoids	Sesquiterpenoids	Aromadendrane sesquiterpenoids	GC-MS + RI + Co-I	Pinto et al. (2016)
159	(−)-aristolene	Terpenoids	Sesquiterpenoids	Aristolane	GC-MS + RI + Co-I	Pinto et al. (2016)
160	α-caryophyllene	Terpenoids	Sesquiterpenoids	Humulane	GC-MS + RI, GC-MS + RI + Co-I	Montes et al. (1988), Di Cosmo et al. (2015), Pinto et al. (2016)
161	Caryophyllene oxide	Terpenoids	Sesquiterpenoids	Caryophyllane		Di Cosmo et al. (2015)
162	α-copaene	Terpenoids	Sesquiterpenoids	Copaane	GC-MS + RI	Niemeyer and Teillier (2007), Di Cosmo et al. (2015)
163	β-patchoulene	Terpenoids	Sesquiterpenoids	Patchoulane sesquiterpenoids	GC-MS + RI	Niemeyer and Teillier (2007)
164	Viridiflorol	Terpenoids	Sesquiterpenoids	Aromadendrane sesquiterpenoids	GC-MS + RI	Di Cosmo et al. (2015), Pinto et al. (2016)

Co-I: co-injection; GC-MS: gas chromatography mass spectrometry, RI: retention index.

**TABLE 5:** Miscellaneous compounds identified in the essential oils from *Cryptocarya alba*.

n°	Compound	Pathway	Superclass	Class	Identification	References
165	2-(1,3-butadienyl) mesitylene	Terpenoids	Miscellaneous	-	GC-MS + RI + Co-I	Pinto et al. (2016)
166	Cinnamic aldehyde	Shikimates and Phenylpropanoids	Phenylpropanoids (C6-C3)	Cinnamic acids and derivatives	GC-MS	Montes et al. (1988)
167	Elemicin	Shikimates and Phenylpropanoids	Phenylpropanoids (C6-C3)	Cinnamic acids and derivatives	GC-MS	Montes et al. (1988)
168	Estragole	Shikimates and Phenylpropanoids	Phenylpropanoids (C6-C3)	Cinnamic acids and derivatives	GC-MS	Montes et al. (1988)
169	Eugenol	Shikimates and Phenylpropanoids	Phenylpropanoids (C6-C3)	Cinnamic acids and derivatives	GC-MS	Montes et al. (1988)
170	Methyleugenol	Shikimates and Phenylpropanoids	Phenylpropanoids (C6-C3)	Cinnamic acids and derivatives	GC-MS + RI	Di Cosmo et al. (2015)
171	Phenyl acetate	Shikimates and Phenylpropanoids	Phenolic acids (C6-C1)	Phenolic acids (C6-C1)	GC-MS	Montes et al. (1988)
172	Phenyl butyrate	Shikimates and Phenylpropanoids	Miscellaneous	-	GC-MS	Montes et al. (1988)
173	Phenylethyl alcohol	Shikimates and Phenylpropanoids	Phenylethanoids (C6-C2)	Phenylethanoids	GC-MS	Montes et al. (1988)
174	β-phenylethyl butyrate	Fatty acids	Fatty esters	Wax monoesters	GC-MS + RI + Co-I	Pinto et al. (2016)
175	Phenylethyl isovalerate	Fatty acids	Fatty esters	Wax monoesters	GC-MS, GC-MS + RI + Co-I	Montes et al. (1988), Pinto et al. (2016)
176	Piperonal	Terpenoids	Monoterpenoids	Menthane monoterpenoids	GC-MS	Montes et al. (1988)
177	Safrole	Shikimates and Phenylpropanoids	Phenylpropanoids (C6-C2)	Cinammic acids and derivatives	GC-MS, GC-MS + RI + Co-I	Montes et al. (1988), Pinto et al. (2016)
178	Veratrol	Shikimates and Phenylpropanoids	Phenylethanoids (C6-C3)	-	GC-MS	Montes et al. (1988)
179	Heptanoic acid	Fatty acids	Fatty acids and conjugates	Brancheda and unsaturated	GC-MS	Montes et al. (1988)
180	Heptanal	Fatty acids	Fatty acyls	Fatty aldehydes	GC-MS + RI, GC-MS + RI + Co-I	Niemeyer and Teillier (2007), Pinto et al. (2016)
181	Heptanol	Fatty acids	Fatty acyls	Fatty alcohols	GC-MS	Montes et al. (1988)
182	Hexanal	Fatty acids	Fatty acyls	Fatty aldehydes	GC-MS	Montes et al. (1988), Di Cosmo et al. (2015)
183	Hexanoic acid	Fatty acids	Fatty acids and conjugates	Fatty acid	GC-MS	Montes et al. (1988)
184	Hexanol	Fatty acids	Fatty acyls	Fatty alcohols	GC-MS	Montes et al. (1988)
185	2-nonanone	Fatty acids	Fatty acyls	Oxygenated hydrocarbon	GC-MS + RI + Co-I	Pinto et al. (2016)
186	2-nonenal	Fatty acids	Fatty acyls	Fatty aldehyde	GC-MS + RI	Di Cosmo et al. (2015)
187	3-octanone	Terpenoids	Monoterpenoids	Iridoids monoterpenoids	GC-MS	Montes et al. (1988)
188	1-octanol	Fatty acids	Fatty acyls	Fatty alcohols	GC-MS	Montes et al. (1988)
189	3-octanol	Fatty acids	Fatty acyls	Fatty alcohols	GC-MS	Montes et al. (1988)
190	1-octen-3-ol	Fatty acids	Fatty acyls	Fatty alcohols	GC-MS	Montes et al. (1988)
191	Pentanol	Fatty acids	Fatty acyls	Fatty alcohols	GC-MS	Montes et al. (1988)
192	*n*-valeraldehyde	Fatty acids	Fatty acyls	Fatty aldehydes	GC-MS	Montes et al. (1988)
193	Isoprenyl isovalerate	Fatty acids	Fatty esters	Wax monoesters	GC-MS + RI	Niemeyer and Teillier (2007)
194	3-methyl-3-butenyl (3-methylbut-3-enyl 3-methylbutanoate) 3-Methyl-3-butenyl isovalerate	Fatty acids	Fatty esters	Wax monoesters	GC-MS + RI	Bravo et al. (2017), Touma et al. (2020)
195	3-methyl-3-buten-1-yl 2-methylbutanoate	Fatty acids	Fatty esters	Wax monoesters	GC-MS + RI	Giordano et al. (2019)
196	Butyric acid	Fatty acids	Fatty Acids and Conjugates	Unsaturated	GC-MS	Montes et al. (1988)
197	Isovaleraldehyde	Fatty acids	Fatty acyls	Fatty aldehydes	GC-MS	Montes et al. (1988)
198	β-apo-13-carotenone	Terpenoids	Apocarotenoids	Apocarotenoids (β-)	GC-MS + RI + Co-I	Pinto et al. (2016)
199	3-decyne	Fatty acids	Fatty acyls	-	GC-MS + RI + Co-I	Pinto et al. (2016)
200	4-(3,3-dimethyl-but-1- ynyl)-4-hydroxy-2,6,6- trimethylcyclohex-2-enone	Terpenoids	Miscellaneous	-	GC-MS + RI + NMR	Di Cosmo et al. (2015)
201	1,5 dimethyl-1,5-cyclooctadiene	Miscellaneous	-	-	GC-MS + RI + Co-I	Pinto et al. (2016)
202	dimethyl-estriol	Terpenoids	Steroids	Estrane	GC-MS	Montes et al. (1988)
203	(5*E*,7*Z*)-5,7-Dodecadien-1-yl acetate	Fatty acids	Fatty esters	Wax monoesters	GC-MS + RI	Giordano et al. (2019)
204	3,9-dodecadiene	Fatty acids	Fatty acyls	Hydrocarbons	GC-MS + RI + Co-I	Pinto et al. (2016)
205	Furfural	Polyketides	Cyclic polyketides	Furans	GC-MS	Montes et al. (1988)
206	3-hexenol	Fatty acids	Fatty acyls	Fatty alcohols	GC-MS + RI	Montes et al. (1988), Di Cosmo et al. (2015)
207	Jasmolin I	Terpenoids	Monoterpenoids	Irregular monoterpenoids	GC-MS + RI + Co-I	Pinto et al. (2016)
208	2-methyl-cyclopentane propanone	Fatty acids	Fatty acyls	Hydrocarbons	GC-MS + RI + Co-I	Pinto et al. (2016)
209	7-octen-2-one	Fatty acids	Fatty acyls	Oxygenated hydrocarbon	GC-MS + RI + Co-I	Pinto et al. (2016)
210	(3*Z*)-2,2,5,5-tetramethyl hex-3-eno	Fatty acids	Fatty esters	Hydrocarbons	GC-MS + RI + Co-I	Pinto et al. (2016)
211	1,3,3-trimethyl-2-(3-methyl-2-methylene-but-3-enylidene)-1-cyclohexanol	Terpenoids	Apocarotenoids	Apocarotenoids (β-)	GC-MS + RI + Co-I	Pinto et al. (2016)

Co-I: co-injection; GC-MS: gas chromatography mass spectrometry, RI: retention index.

### 3.2 Phytochemistry of the fruit of *Cryptocarya alba*

The fruit of *C. alba* showed variations in its chemical composition. The peel contains 5.6% moisture, while the pulp and seed have 5.3%. For crude protein, the peel contains 6.88%, which is slightly higher than the 6.25% found in the pulp and seed. The crude lipid content is much higher in the peel (32.30%) compared to the pulp and seed (16.15%). Crude fiber also differs significantly, with 20.40% in the peel versus 5.60% in the pulp and seed (Schmeda-Hirschmann et al., 1999). Regarding ash, the peel has 1.80%, while the pulp and seed contain 2.30%. The nitrogen-free extract is higher in the pulp and seed (69.70%) than in the peel (38.62%). Finally, the phosphorus content is similar in both, with 124 mg in the peel and 130 mg in the pulp and seed (Schmeda-Hirschmann et al., 1999). The fatty acid composition differs between the fruit and its peel. In the fruit, palmitic acid (16:0) accounts for 15.73% of the total fatty acids, whereas in the peel, it is lower at 10.00%. Palmitoleic acid (16:1) is more abundant in the peel (17.15%) than in the fruit (9.51%). Stearic acid (18:0) shows similar levels in both, with 2.18% in the fruit and 1.52% in the peel. Oleic acid (18:1) is the most prevalent fatty acid in both parts, with 44.50% in the fruit and 56.92% in the peel. Linoleic acid (18:2) is found in higher amounts in the fruit (25.03%) compared to the peel (9.51%), while linolenic acid (18:3) shows similar levels, with 2.93% in the fruit and 3.23% in the peel (Schmeda-Hirschmann et al., 1999).

#### 3.2.1 Polyphenols in the fruit of *Cryptocarya alba*

Furthermore, the fruit contains methyl (4-caffeoyl)-quinate, quercetin-3-*O*-pentoside, luteolin 8-*C*-glucoside (orientin), and 8-methoxy-kaempferol (Simirgiotis, 2013). Additionally, several bioactive compounds were identified in *C. alba* fruit, including polyphenolic acids, quercetin derivatives, procyanidins, catechin, epicatechin, chlorogenic acid, and an alkaloid (Valdenegro et al., 2021). This work tentatively identified by UHPLC/MS analysis (+)-cryptorigidifoliol A, an α,β-unsaturated δ-lactone found in *Cryptocarya* species, isolated from *C. rigidifolia*, and showing antiparasitic, antimycobacterial, antitumor, anticancer, and antimalarial activities (Raju et al., 2015). Regarding fruit pigments, after study, it was not possible to find anthocyanins or carotenoids in detectable amounts in *C. alba* fruits. The peel color of this species may be produced by tannins or a combination of other compounds detected in this species; the structures of the tentatively identified compounds by MS spectra included quinic acid, malvidin-3-*O*-(4‴-coumaroyl)-rutinoside-5-*O*-glucoside, peonidin-3-*O*-(4‴-coumaroyl)-rutinoside-5-*O*-glucoside, malvidin-3-*O*-(4‴-coumaroyl)-rutinoside, and importantly petunidin-3-*O*-(4‴-coumaroyl)-rutinoside-5-*O*-glucoside (Simirgiotis, 2013).

#### 3.2.2 Other metabolites in the fruit of *Cryptocarya alba*

In the methanolic extract of *C. alba* fruit, a lignan (+)-lariciresinol, also found in *Cryptocarya impressinervia* (Viktorová et al., 2020; Valdenegro et al., 2021; Xiong et al., 2021), and other compounds specific to the genus *Cryptocarya,* such as (−)-rubrichalcolactone, cryptorigidifoliol A, and isocryprochine or cryprocine (Cavalheiro and Yoshida, 2000), have been tentatively identified. Regarding the first, (−)-rubrichalcolactone was found only in a dichloromethane fraction of an ethanol-soluble extract of the leaves and twigs of *Cryptocarya rubra* (Ren et al., 2014). Cryptorigidifoliol A has been isolated from the root wood of *Cryptocarya rigidifolia* (Liu et al., 2015).

The genus *Cryptocarya* includes species that produce α-pyrones, such as cryptofolione, thereby increasing its chemical diversity and biological potential (Cavalheiro and Yoshida, 2000). Cryptofolione was extracted from the fruits of *C. alba* at a 0.015% yield (Schmeda-Hirschmann et al., 2001). This compound has previously been identified in other African *Cryptocarya* species, marking the first time it was found in *C. latifolia* and *C. myrtifolia*, both of which are used in traditional Zulu medicine to treat respiratory conditions and for ritual purposes (Sehlapelo et al., 1994). Cryptofolione has been detected at trace levels in *C. wyliei* and *C. woodii* (Drewes et al., 1995), *C. liebertiana* (Drewes et al., 1997), and *C. concinna* (Sturgeon et al., 2008). In Brazilian *Cryptocarya*, such as *Cryptocarya moschata* (or *Cryptocarya mandioccana*), cryptofolione is a key secondary metabolite used to distinguish *C. mandioccana* chemotypes (Cavalheiro and Yoshida, 2000), with its production influenced by both genetic and environmental factors (Nehme et al., 2008).

## 4 Traditional, cultural, and ethnopharmacological uses of *Cryptocarya alba* in indigenous communities of Central-Southern Chile

Archaeological evidence from Los Catalanes Cave in southern Chile shows that *C. alba* was an important food source for prehistoric peoples during the Early Ceramic Period (ECP, 350–1000 AD). This tree species, known for its edible fruits, was part of a plant resource collection that also included maqui (*Aristotelia chilensis* (Molina) Stuntz [Elaeocarpaceae]) and, possibly, the native hazelnut (*Gevuina avellana* Molina [Proteaceae]). The presence of these remains in the stratigraphic units linked to the ECP indicates they were consistently used throughout this period (Roa Solís et al., 2024). Chemical analysis of residues found in pipe mouthpieces and bowls at the La Granja archaeological site in Chile’s Central Valley, associated with the prehistoric cultural complex (500–1000 AD), detected residues of wild tobacco (*Nicotiana* sp.) along with 14 other species, including *C. alba*, suggesting they were smoked together in a mixture (Planella et al., 2016). Studies analyzing microfossils from dental calculus at the Villa Virginia archaeological site (ECP) provide evidence of *C. alba* fruit consumption showing signs of thermal alteration (Ramírez-Funes et al., 2023). Archaeological finds at the Late Early Period (LEP)-C site, a well-studied coastal Llolleo culture settlement in Chile, support these insights into the ancient use of plants. Here, dietary evidence shows a heavy reliance on marine resources, including mollusks and fish. Notably, remains of wild edible C3 plants, such as *C. alba* and the Chilean palm coconut (*Jubaea chilensis* (Molina) Baill. [Arecaceae]), were also found, alongside land and lagoon fauna. This coastal diet, seen across the pre-ceramic and agro-pottery phases of the region (Falabella and Planella, 1991), complements findings from Mocha Island, reinforcing the widespread presence and potential management of *C. alba* by pre-Hispanic communities in Chile. An analysis of charcoal at three archaeological sites on Mocha Island (850–1685 AD) revealed that *C. alba* was present during pre-Hispanic times, despite its absence in later botanical records. The study suggests the island had a mixed forest of lauriphyllous and sclerophyllous trees, indicating neither dense forest nor clear-cut landscapes. Inhabitants likely maintained ecotonal zones, possibly to cultivate useful species like *C. alba* (Delgado-Orellana, 2025).

At nearby continental archaeological sites such as Purén and Lumaco, during a similar period, the Late Pre-Hispanic El Vergel period in Central-Southern Chile is marked by significant cultural shifts. Archaeological evidence indicates the rise of mound-building and a broad-spectrum subsistence strategy that includes hunting, gathering, and horticulture. Examining domestic sites in Purén and Lumaco reveals the consumption of various plants, including domesticated species like *Solanum tuberosum* L. [Solanaceae], *Zea mays* L. [Poaceae], and *Chenopodium quinoa* Willd. [Amaranthaceae], alongside wild foods such as *C. alba* and *Fragaria chiloensis* (L.) Mill. [Rosaceae] (Dillehay, 2007). *C. alba* has a strong historical link to ethnomedicine. Records show that its bark, leaves, and fruits have been used in infusions, baths, and poultices (Castro-Saavedra et al., 2016b), practices that were mainly abandoned by local communities. However, they still consume infusions of boldo (*Peumus boldus* Molina [Monimiaceae], bailahuén (*Haplopappus* sp. [Asteraceae]), and matico (*Buddleja globosa* Hope [Scrophulariaceae]) for medicinal purposes (Burgos and Morales, 2010; Madaleno and Delatorre-Herrera, 2013; Leighton and Monsalve, 2015; Bridi et al., 2023). The bark and leaves of *C. alba* are traditionally known as rich sources of tannins (Castro-Saavedra et al., 2016b). Internally, an aqueous extract of the leaves has been used for liver issues and vaginal bleeding (Muñoz Schick and Barrera, 1981). Externally, a decoction of the bark and/or leaves is employed to relieve rheumatism symptoms (De Moesbach, 1992). Another preparation involves infusing the leaves *of C. alba* in wine or an alcohol tincture, which is then applied to the affected limbs and other areas (Muñoz Schick and Barrera, 1981). Ground seeds of *C. alba* are used to make ointments for vaginal infections and for abdominal problems associated with colds. A liquid extract from the plant is administered vaginally to stop bleeding and treat leucorrhea (Muñoz Schick and Barrera, 1981). The fruits of *C. alba* are aromatic and edible, but they need to be cooked or infused to remove their bitterness. They can also be eaten raw by holding them in the mouth so saliva neutralizes the bitter taste. Today, this tree is considered a non-timber forest product, defined as a source of goods of biological origin other than wood. Some researchers believe that its use as food and in traditional medicine has increased in recent decades (Simirgiotis, 2013; Giordano et al., 2019; Peña-Rojas et al., 2021). Peumo biomass is used to produce shampoos, cosmetics, beer, and other food products, indicating an informal market for local people (Simirgiotis, 2013). The Huilliche people of Chile have used the EOs from aromatic species like *C. alba* to treat wounds and related infections (Bravo, 2021).

## 5 Human health-related bioactivity and toxicity

### 5.1 Antibacterial activity

The ethyl acetate extract of *C. alba* leaves was inactive against the Gram-negative bacterium *Chromobacterium violaceum* at 100 μg/disc (Carcamo et al., 2014). However, the EO of *C. alba* has been studied in several trials. For instance, it showed mild antibacterial activity against *Staphylococcus aureus* (inhibition zone diameter of 6–9 mm) using the well method (Avello Lorca et al., 2012). The EO from *C. alba* leaves was effective against *S. aureus* (25 mm inhibition zone), *E. coli* (8 mm inhibition zone), and *H. pylori*. The minimum inhibitory concentration (MIC) values of the EO were 19.0 μg/mL against *S. aureus*, 36.0 μg/mL against *Escherichia coli*, and 30.0 μg/mL against *Helicobacter pylori* (Touma et al., 2020). In this context, the main components of the EO demonstrated their effectiveness: alpha-terpineol (MIC values: 27 μg/mL against *H. pylori*, 32 μg/mL against *S. aureus*, 16 μg/mL against *E. coli*), eucalyptol (MIC: 30 μg/mL against *H. pylori*, 32 μg/mL against *S. aureus*, 32 μg/mL against *E. coli*), and beta-phellandrene (MIC: 30 μg/mL against *H. pylori*, 32 μg/mL against *S. aureus*, 32 μg/mL against *E. coli*). Alpha-terpineol was especially effective against *H. pylori* and *E. coli*, exhibiting the lowest MIC values among the other compounds. Eucalyptol and beta-phellandrene also exhibited antimicrobial activity, though to a lesser extent (Touma et al., 2020).

The methanolic extract from the fruit of *C. alba* showed limited ability to inhibit *S. aureus* strains, with IC_50_ values of 0.533 ± 0.018 mg/mL for the sensitive strain and 0.557 ± 0.034 mg/mL for the resistant strain, indicating no selectivity between them (Viktorová et al., 2020). However, the same extract demonstrated inhibitory activity against bacterial quorum sensing, with varying effectiveness across strains. For the Gram-negative strain A1-12 BAA 1118 (G-), a concentration of 165.7 ± 21.8 μg/mL was needed to inhibit 50% of its viability, while only 25.0 ± 0.8 μg/mL was enough to inhibit 50% of its communication. Conversely, the IC_50_ values for the A1-2 BAA 1116 strain were 147.4 ± 2.3 μg/mL for viability and 98.2 ± 7.4 μg/mL for communication. These findings suggest that *C. alba* extract has a strong ability to disrupt bacterial communication, particularly in Gram-negative strains, at concentrations lower than those required to inhibit growth or survival (Viktorová et al., 2020).

### 5.2 Antioxidant capacity

*Cryptocarya alba* leaf extracts contain 1263 μg of chlorogenic acid equivalents per gram (CAE/g) (Simirgiotis, 2013). However, characterizing the polyphenol content in peumo is quite complex. The most notable component is the leaves, with content varying significantly between individuals, ranging from 54 to 131 mg of gallic acid equivalents (GAE) per gram of dry weight (DW). Similarly, the flavonoid content ranges from 8.5 to 21.9 mg of quercetin equivalents (QE) per gram of DW. In terms of distribution, the bark shows slightly lower levels, while the wood shows considerably lower levels. Regarding antioxidant capacity, the 2,2′-azino-bis-(3-ethylbenzothiazoline-6-sulfononic acid) diammonium salt (ABTS) and 2,2-Diphenyl-1-picrylhydrazyl (DPPH) assays show a similar trend; however, when assessed using the ferric reducing antioxidant potential (FRAP) method, the bark exhibits higher values. All of these variables exhibit high variability at both the individual and population levels (Giordano et al., 2019).

Another study, based on a small number of leaf samples, reports an antioxidant activity of 98.3 μmol of Trolox equivalents per gram of fresh weight (TE/g FW) (Simirgiotis, 2013). The overall analysis of the methanolic extracts from *C. alba* fruits and aerial parts showed a total polyphenol content of 17.70 mg GAE/g. Regarding antioxidant activity, the fruits demonstrated a remarkable ability to neutralize the DPPH radical, with an IC_50_ value of 9.12 μg/mL and a FRAP value of 39.65 μmol TE/g (Simirgiotis, 2013). Furthermore, at a concentration of 50 μg/mL, the methanolic extract of the fruits inhibited 70% of the DPPH radical, highlighting its antioxidant potential (Schmeda-Hirschmann et al., 1999). The ripe fruit exhibited a high total polyphenol content, reaching approximately 17.61 mg GAE/g FW, which is significantly higher than that of ripe blueberries (2.75 mg GAE/g FW). Additionally, it demonstrated outstanding antioxidant capacity across various assays, including FRAP (37.08 µmol FeSO_4_/g FW), TEAC (7.91 mmol TE/g FW), DPPH (IC_50_ of 8.35 μg/mL), and ORAC (0.188 mmol TE/g FW) (Valdenegro et al., 2021). The EOs of *C. alba* leaves contain 163.6 ± 10.7 mg GAE/g of phenolics and have a FRAP reducing capacity of 166.8 ± 27.9 mg TE/g. It exhibits moderate DPPH radical scavenging activity (IC_50_ = 417.8 ± 5.8 μg/mL) and greater ability to inhibit the ABTS radical (IC_50_ = 203.0 ± 12.8 μg/mL). According to the authors, the EOs' overall antioxidant activity is attributed to their rich terpene composition, which includes conjugated hexadiene structures and hydroxylated terpenes, such as alpha-terpineol. These compounds enable it to function effectively as an electron donor and free radical scavenger, contributing to its potent antioxidant effect (Touma et al., 2020).

### 5.3 Other biological activities

#### 5.3.1 Enzymatic inhibition

Leaf and bark extracts of *C. alba* showed a low ability to inhibit xanthine oxidase (XO). Regarding inhibition of beta-glucuronidase, the leaf extract had an IC_50_ value of 7 μg/mL, whereas the bark extract was more potent, with an IC_50_ value below 4 μg/mL (Schmeda-Hirschmann et al., 1992). Conversely, the methanolic extract from the fruits inhibited XO by 32%, a key enzyme in the treatment of gout and hyperuricemia. However, its effect on beta-glucuronidase was limited, reaching only 5% inhibition at 50 μg/mL (Schmeda-Hirschmann et al., 1999). In addition, the methanolic extract of the leaves has been tested as a 15-lipoxygenase inhibitor and found to be inactive (IC_50_ > 200 μg/mL) (Castro-Saavedra et al., 2016a).

#### 5.3.2 Anti-inflammatory effect

The methanolic extract of *C. alba* reduced the production of three inflammatory markers in lipopolysaccharide (LPS)-stimulated macrophages (RAW 264.7): Nitric oxide (NO) (IC_50_ of 13.2 ± 0.5 mg/L), tumor necrosis factor-alpha (TNF-α) (IC_50_ of 129.5 ± 3.5 mg/L), and interleukin-6 (IL-6) (IC_50_ of 40.0 ± 4.0 mg/L). Compared to quercetin, *C. alba* was less effective at inhibiting these markers. However, relative to indomethacin, *C. alba* was more efficient at inhibiting NO production but less effective at reducing TNF-α and IL-6 levels (Valdenegro et al., 2021).

#### 5.3.3 Vasoprotective effect

Regarding its hypotensive effect, the leaf extract did not change blood pressure in rats (Schmeda-Hirschmann et al., 1992). However, the fruit extract demonstrated protection of endothelial function. Specifically, there was a partial reversal of the endothelium-dependent relaxation impairment, with significant differences observed at concentrations of 1 and 10 mg/mL. Nonetheless, the pD2 value of acetylcholine (ACh) did not change significantly across the tested extract concentrations, suggesting that *C. alba* exerts its vasoprotective effects through ACh-independent mechanisms (Valdenegro et al., 2021).

#### 5.3.4 Antiproliferative effects on cancer cell lines

The ethanolic extract of *C. alba* leaves showed cytotoxic activity against mammary adenocarcinoma cells (MCF-7), with an IC_50_ value of 73.28 ± 4.75 μg/mL. At the same time, it displayed relatively low toxicity in non-tumor cells (MCF10A), with an IC_50_ value of 132.63 ± 4.77 μg/mL. This suggests potential selectivity of the extract toward tumor cells, although its cytotoxic effect remains moderate (Castro-Saavedra et al., 2016a). The EO of *C. alba* demonstrates a diverse and selective biological activity profile. It inhibits the growth of MCF-7 mammary tumor cells while sparing the viability of non-tumor MCF-10A mammary epithelial cells. Additionally, it shows low toxicity toward healthy HK2 kidney cells. The EO has a potent antiproliferative effect on 786-O renal cell carcinoma, with a lesser impact on metastatic ACHN renal cell carcinoma. In U87MG glioblastoma cells and human fibroblasts, the inhibition was concentration-dependent. The inhibition observed in fibroblasts warrants careful evaluation of its effects on non-pathological tissues (Touma et al., 2020).

#### 5.3.5 Activity against human pathogenic fungi

The EO demonstrated antifungal activity against *Candida albicans*, with an MIC value of 31 μg/mL. Among the main components of the EO, alpha-terpineol exhibited the same MIC value of 16 μg/mL against *C. albicans*. At the same time, beta-phellandrene and eucalyptol had MIC values of 32 μg/mL each (Touma et al., 2020).

#### 5.3.6 Trypanocidal activity

Cryptofolione, isolated from the fruits of *C. alba*, showed significant trypanocidal activity (77% reduction of *Trypanosoma cruzi* parasites at 250 μg/mL) and moderate leishmanicidal activity (about 70% lysis of promastigotes), along with moderate cytotoxicity in macrophages, which limits its therapeutic potential (Schmeda-Hirschmann et al., 2001). Extracts of dichloromethane and methanol/water from the unspecified plant parts were tested against trypomastigotes at concentrations up to 500 μg/mL and found to be inactive (Muñoz et al., 2013).

#### 5.3.7 Antimutagenic and mutagenic activity

Mutagens are chemical or physical agents that can modify genetic material, increasing the risk of cancer and other diseases. Antimutagens are substances or agents that decrease the frequency of DNA mutations, either by preventing their formation or by facilitating the repair of genetic damage. At 0.50 mg/mL, the fruit extract of *C. alba* showed 57% DNA binding, indicating the presence of bioactive compounds capable of interacting with genetic material (Schmeda-Hirschmann et al., 1999). In this context, the leaf extract of *C. alba* at concentrations between 4.74 and 9.49 mg/mL exhibited a desmutagenic effect by reducing single spots—the result of mutations—and twin spots (arising from mitotic recombination), as well as the total number of spots. However, it did not affect large spots. The extract contained numerous metabolites including quercitrin, chlorogenic acid, and kaempferol-3-*O*-β-galactoside, as reported by (Timmermann et al., 1995), along with anthocyanins such as cyanidin, peonidin, and malvidin (Carmona et al., 2017). The aqueous leaf extract combined with the mutagenic agent ethyl methanesulfonate (EMS) demonstrated a significant reduction in various mutant spot types compared to EMS alone. In *Drosophila melanogaster*, *C. alba* extract did not induce mutagenicity, as it did not increase the frequency of mutant spots on wings (Carmona et al., 2017).

#### 5.3.8 Evaluation of toxicity in animal models

The *Artemia salina* assay was used to assess the overall toxicity of hydroalcoholic extracts from *C. alba*. The leaf extract had a lethal concentration for 50% of the organisms (LC_50_) of 253 μg/mL, while the bark extract showed much higher LC_50_ values of 2071 μg/mL (Schmeda-Hirschmann et al., 1992). The EO of *C. alba* exhibited low or no toxicity against the nematode *Caenorhabditis elegans* at concentrations from 0.39 to 50 mg/mL (Touma et al., 2020). The median lethal dose (LD_50_) of reticuline (the most critical alkaloid) when administered intraperitoneally (i.p.) to mice and rats was 251 mg/kg and 216 mg/kg, respectively (Morais et al., 1998).

## 6 Non-human health-related bioactivity and toxicity

### 6.1 Insecticidal activity

*Cryptocarya alba* EO shows insecticidal activity against *Sitophilus zeamais*. The highest mortality, 94%, occurred at a concentration of 80 mL of EO per kg of grain. The estimated LC_50_ was 14.6 mL EO/kg, indicating that relatively high concentrations are required to produce a significant effect. The primary mechanism is likely ovicidal, with compounds like 1,8-cineole and terpineol contributing to toxicity due to their known insecticidal properties (Pinto et al., 2016). *C. alba* EO also demonstrated insecticidal activity against the house fly (*Musca domestica*), with an LD_50_ of 33.56 mg/dm^3^ at 0.5 h and 15.07 mg/dm^3^ at 1 h (at 26 °C ± 1 °C), indicating increased effectiveness over time. Among the compounds, 1,8-cineole had the highest insecticidal potency (LC_50_ value of 3.35 mg/dm^3^ at 0.5 h), followed by alpha-pinene (12.1 mg/dm^3^) and 4-terpineol (36.8 mg/dm^3^), indicating this EO’s potential as a natural insecticide (Di Cosmo et al., 2015).

### 6.2 Activity against phyto/entomopathogenic fungi

The EO of *C. alba* induced a significant morphological change in *Penicillium* sp., resulting in the formation of sclerotia (resistance structures). In contrast, *Fusarium oxysporum* showed no effect from the EO but did exhibit morphological changes in mycelial growth at a 2% concentration of the oil. In vapor phase exposure, no antifungal activity was observed against *Penicillium* sp. or *F. oxysporum* at any concentrations tested (Avello Lorca et al., 2012).

On the other hand, in the field of agricultural pathogens, the EO of *C. alba* leaves has been shown to have antifungal activity against *Nosema ceranae* at a concentration of 4 µg/bee. This is a unicellular microsporidian fungus that parasitizes honeybees (*Apis mellifera*). The main compounds identified in this EO—alpha-terpineol, eucalyptol, and beta-phellandrene—demonstrated significant effects in controlling the fungus. However, the antifungal activity of the complete EO was greater than that observed with each of these isolated compounds. This suggests that the EOs could be a promising candidate for the treatment or prevention of nosemosis in bees (Bravo et al., 2017).

## 7 Pharmacological effects of the main phytochemicals of *Cryptocarya alba*

The medicinal properties of the species are linked to compounds such as chlorogenic acid, epicatechin, quercitrin, rutin, procyanidins, and reticuline, which are mainly found in the aboveground biomass of *C. alba* (Giordano et al., 2019; Antileo-Laurie et al., 2023). Due to their quantity and biological activity, these compounds could serve as the active ingredients in dried medicinal plants. Currently, their medicinal properties and uses in the food industry are being researched for potential health benefits (Simirgiotis, 2013; Giordano et al., 2019; Antileo-Laurie et al., 2023).

### 7.1 Chlorogenic acid

Chlorogenic acid (CGA) is a naturally occurring polyphenolic compound abundant in various plants. It is known for its strong antioxidant properties and multiple health benefits, including neuroprotection, modulation of inflammation and oxidation, and support of metabolic balance (Heitman and Ingram, 2017; Wang et al., 2022). Its effects span several areas, including protection against neurodegenerative disorders and diabetic neuropathy, reduced risk of cardiovascular, skin, liver, and kidney diseases, and significant antitumor activity (Nguyen et al., 2024). Mechanistically, CGA influences key pathways, including nuclear factor kappa-light-chain-enhancer of activated B cells (NF-κB), nuclear factor erythroid 2-related factor 2 (Nrf2), and AMP-activated protein kinase (AMPK), reducing inflammation, oxidative stress, and metabolic disturbances. Additionally, it affects neuronal activity through interactions with neuroreceptors and ion channels (Nguyen et al., 2024). Besides its health benefits, CGA has various applications in the food industry, where it serves as an additive, preservative, and functional food enhancer. Its prebiotic potential has sparked increasing research interest (Ochiai et al., 2019; Wang et al., 2022).

### 7.2 Epicatechin and procyanidins

Epicatechin (EC) and procyanidins are additional important phytochemicals in *C. alba*. These natural polyphenols, found in sources such as grapes, cocoa, coconut, boldo, and apples, are known for their strong antioxidant activity and varied properties, which have spurred research into natural sources (Urpi-Sarda et al., 2009; Pastene et al., 2014; Keivani et al., 2024).

EC increases antioxidant levels in human plasma and improves endothelial function (Cremonini et al., 2020) and has emerged as a safe and promising therapeutic candidate for treating metabolic diseases (Abdulkhaleq et al., 2017). Furthermore, it inhibits platelet aggregation, a beneficial effect for cardiovascular health. Its ability to reduce insulin resistance makes it a promising compound for the treatment of type II diabetes (Cremonini et al., 2020). EC exerts beneficial effects on skeletal muscle, including reducing fibrosis (Ramirez-Sanchez et al., 2014), improving muscle function, inducing mitochondrial biogenesis (McDonald et al., 2021), and enhancing tissue repair (Ramírez-Ramírez et al., 2022). Additionally, EC has shown potential to mitigate and delay muscle loss in musculoskeletal diseases, such as sarcopenia and muscle atrophy. This is due to its ability to regulate muscle growth via the insulin-like growth factor (IGF)-phosphatidylinositol 3-kinase (PI3K)-protein kinase B (AKT) pathway, stimulate protein synthesis, and reduce catabolic effects (German et al., 2024). A systematic review of scientific literature (German et al., 2024) identified a strong evidence on the effects of EC in regulating atrogens' expression and activating key myogenic regulatory factors. The findings suggest that exercise training promotes AKT/mammalian target of rapamycin (mTOR) signaling and stimulates mitochondrial synthesis. In a maternal obesogenic environment, EC acts as a specific modulator of myomiRNA expression in offspring, with effects depending on the muscle type analyzed. Treatment with EC consistently reduced miRNA-31-5p expression in both the gastrocnemius and soleus muscles, regardless of maternal condition (control or obese). EC also prevented the increase in miRNA-296 expression caused by the obesogenic environment in both muscles. Conversely, in the soleus muscle of offspring from obese mothers, EC decreased miRNA-486 expression, while in the gastrocnemius muscle of offspring from control mothers, it increased this same miRNA’s expression (Zárate-Segura et al., 2025).

Numerous studies support the potential of procyanidins in managing metabolic and inflammatory diseases due to their strong antioxidant properties. Procyanidins surpass the antioxidant capacity of vitamins C and E, protecting against oxidative stress caused by reactive oxygen species (ROS) (Rauf et al., 2019). By neutralizing ROS and reactive nitrogen species (RNS), they prevent damage to DNA, lipids, and proteins, reducing the risk of diseases such as cancer, neurodegenerative disorders, and cardiovascular conditions (Dasiman et al., 2022). Additionally, procyanidins promote DNA repair, regulate stress signaling pathways and apoptosis, and boost the activity of antioxidant enzymes (Rauf et al., 2019). They lower lipid peroxidation, guard against heavy metal-induced ROS production, and influence nitric oxide production and the release of proinflammatory cytokines. They also affect lipid metabolism by reducing lipid and cholesterol absorption (Dasiman et al., 2022). Pycnogenol®, a bark extract from maritime pine (*Pinus pinaster* Aiton [Pinaceae]) that has been marketed since the mid-20th century, consists of 58% monomers and dimers of catechin and EC (D’andrea, 2010), compounds similar to those found in *C. alba* bark (Giordano et al., 2019). With strong scientific backing supporting its use as a nutritional supplement and phytopharmaceutical, Pycnogenol® has shown effectiveness in alleviating conditions related to oxidative stress, inflammation, and circulatory issues (D’andrea, 2010; Iravani and Zolfaghari, 2011). Other well-supported maritime pine bark extracts include Flavangenol®, Enzogenol®, and Oligopin® (Robertson et al., 2020), which have demonstrated promising results in women’s health. In two double-blind, randomized clinical trials, Oligopin® showed beneficial effects in postmenopausal women with osteopenia, increasing bone formation markers and decreasing bone resorption (Majidi et al., 2021), as well as improving osteocalcin levels, the osteocalcin (OC)/type I collagen cross-linked C-telopeptide (CTX-1) ratio, oxidative stress, and antioxidant capacity (Panahande et al., 2019).

### 7.3 Reticuline

Administering reticuline produces depressant effects on the central nervous system (CNS), as evidenced by inhibition of locomotor activity (Kimura et al., 1983). At a dose of 25 μg, it temporarily suppresses spontaneous locomotor activity for about 15 min while still allowing responsiveness to external stimuli. At 100 μg, it causes severe immobility, cataleptic posture, Straub’s tail reaction, and reduced responses to tactile and noxious stimuli (Watanabe et al., 1981). In terms of its interaction with the dopaminergic system, reticuline acts as an antagonist of apomorphine-induced behaviors, particularly contralateral rotational responses in 6-hydroxydopamine (6-OHDA)-lesioned mice, at doses of 40 and 80 µg. However, even at 100 μg, it does not affect methamphetamine-induced hyperactivity or ipsilateral rotational behavior, indicating a primarily postsynaptic mechanism of action (Watanabe et al., 1981). These results suggest that reticuline exerts a depressant effect on the CNS, evidenced by the prolongation of pentobarbital-induced sleep, decreased locomotor and exploratory behaviors, and impaired motor coordination. Additionally, the effects seen in the active avoidance test, along with the inhibition of amphetamine-induced hyperactivity, imply possible dopaminergic antagonist activity. These findings support reticuline’s potential as a modulator of the dopaminergic system, with possible implications for the development of naturally derived neuroleptic agents (Morais et al., 1998). Reticuline, found in the alkaloidal-rich fraction (AFDF) of *Duguetia furfuracea* (A.St.-Hil.) Saff. [Annonaceae] has notable effects on the CNS. Recent studies have shown that AFDF exhibits anxiolytic activity and reduces scopolamine-induced memory impairment (Fava de Souza et al., 2024). In the open field test, oral administration of AFDF (30 mg/kg) increased time spent in the central zone by 80% (*p* < 0.01) and decreased rearing by 69% (*p* < 0.01), indicating an anxiolytic effect. Additionally, AFDF decreased grooming by 70% (*p* < 0.001), with no significant differences compared to diazepam (DZP, 2 mg/kg) (Fava de Souza et al., 2024). In the scopolamine-induced spatial memory impairment model, AFDF effectively reversed the deficit, improving spatial learning and memory with effects comparable to those of donepezil. These findings suggest that reticuline-rich alkaloidal extracts could be promising agents for the treatment of neurocognitive disorders (Fava de Souza et al., 2024).

Moreover, AFDF reduced LPS-induced neuroinflammation in mice by decreasing microglial activation and levels of brain inflammatory markers. It also alleviated pathological changes and improved learning and memory impairments associated with neuroinflammation (Fava de Souza et al., 2024). Similarly, neuroinflammation was induced in C57BL/6J mice by administering LPS intraperitoneally for 14 days. The effects of the ethanolic extract on cognition were assessed using spontaneous activity tests, object recognition, and the Morris water maze. Histopathological changes in the hippocampus, along with levels of inflammatory genes and proteins (measured via quantitative real-time polymerase chain reaction (PCR) and enzyme-linked immunosorbent assay), and microglial activation were examined. Lastly, network pharmacology was employed to predict the targets and pathways affected by the plasma components of *Tinospora sinensis* (Lour.) Merr. [Menispermaceae], identifying six compounds, including reticuline, in plasma responsible for the activity (Xie et al., 2025).

Reticuline shows antispasmodic and neuromuscular blocking effects. It functions as an antagonist of acetylcholine- and calcium-induced contractions in uterine muscle and inhibits potassium-induced contractions in the *vas deferens*, with greater effectiveness noted during the tonic phase. Its mechanism of action is likely to involve calcium antagonism (Martin et al., 1993).

Reticuline exhibits anti-inflammatory effects in animal models. In mice, doses of 0.25 mg/kg and 0.5 mg/kg significantly reduced xylene-induced ear swelling. In rats, a 0.5 mg/kg dose decreased carrageenan-induced paw swelling 1–3 h after injection. Additionally, reticuline suppressed the expression of TNF-α and IL-6, which encode proinflammatory cytokines, and lowered the phosphorylation levels of Janus kinase 2 (JAK2) and signal transducer and activator of transcription 3 (STAT3) proteins involved in inflammatory signaling (Yang et al., 2018). Reticuline reduced airway resistance, decreased inflammatory infiltration in lung tissue, and lessened the recruitment of inflammatory cells in bronchoalveolar lavage fluid in obese mice with induced asthma. It also lowered levels of the interleukins IL-17A, IL-1β, and IL-5, as well as macrophage inflammatory protein 2, and increased the number of normal T cells. Reticuline inactivates the JAK2/STAT3/suppressor of cytokine signaling-3 (SOCS3) and p38 subgroup of mitogen-activated protein kinases (MAPKs)/NF-κB signaling pathways in obesity-related asthma (Lyu et al., 2024).

In normotensive rats, acute intravenous administration of reticuline (5–20 mg/kg) causes significant hypotension. In isolated aortic rings, reticuline (3 × 10^−6^ to 1.5 × 10^−3^ M) inhibits contractions induced by phenylephrine and KCl (30 and 80 mM), both with and without endothelium. However, the inhibitory effect is more prominent when the endothelium is intact, indicating that endothelial factors enhance its vasorelaxant action (Dias et al., 2004).

**FIGURE 14:**
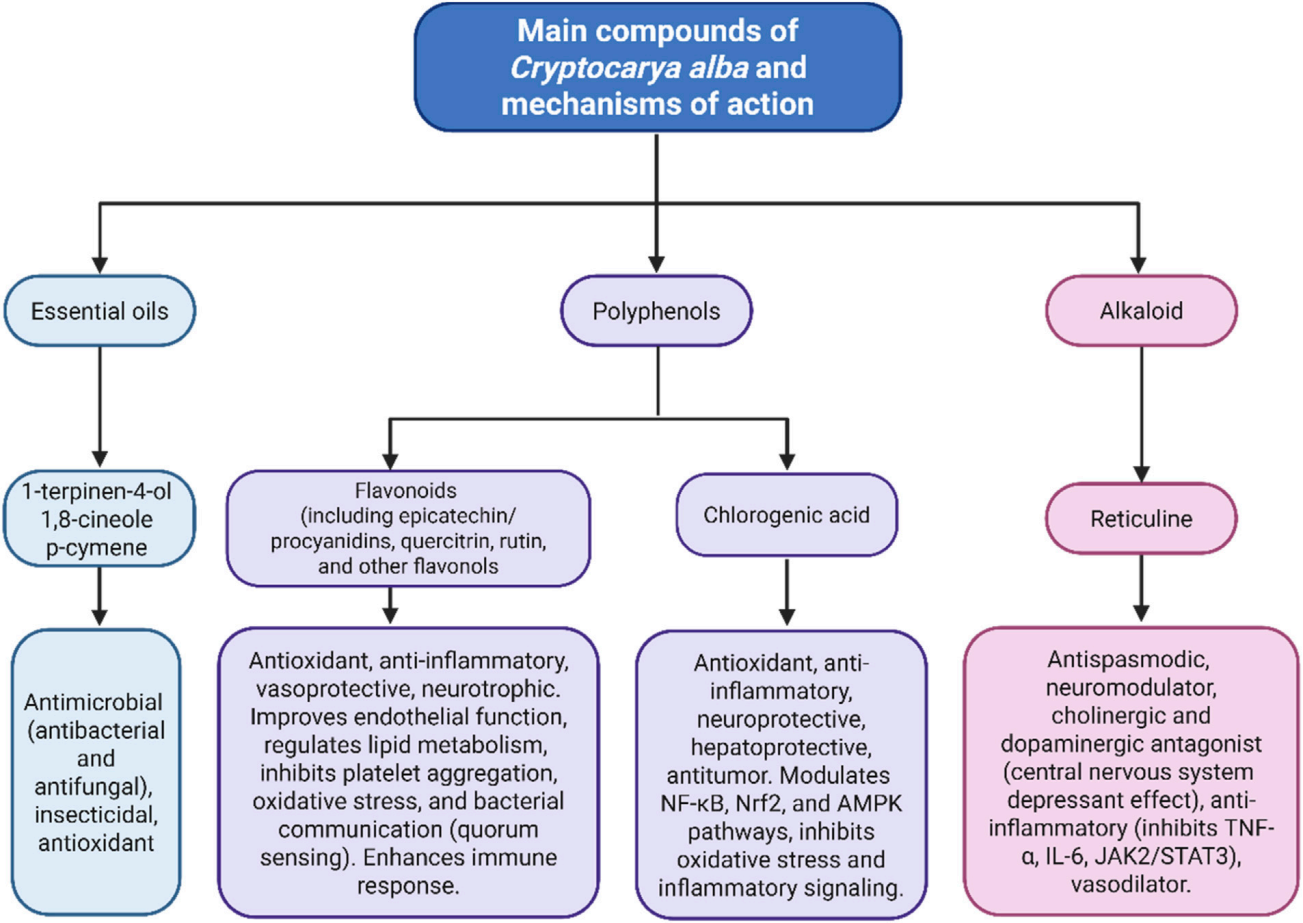
Main compounds of *Cryptocarya alba* and their pharmacological activities and mechanisms of action.

Figure 14 summarizes the pharmacological activities and mechanisms of action of the primary compounds identified in *C. alba*.

## 8 Ecological aspects of chemical variation

The chemical composition of individuals within the same species can vary due to factors such as developmental stage, climate conditions, and soil nutrient availability. (Gobbo-Neto and Lopes, 2007; Nehme et al., 2008; Fuentes-Barros et al., 2018; Da Silva Antonio et al., 2024).

In the bark of very long-lived *C. alba* trees, negligible amounts of chlorogenic acid, catechin, quercetin, epicatechin, and procyanidins (B1, B2, and C1) were found compared to the bark of younger trees (Giordano et al., 2019). The opposite was true for leaves: leaves from older trees had higher concentrations of chlorogenic acid, quercetin, and quercitrin, and no individual exhibited isorhamnetin (Giordano et al., 2019). Among leaves from the same tree, age was also associated with a profile, with older leaves showing higher levels of quercetin, procyanidins, quercitrin, and cryptochlorogenic acid. However, the differences in chlorogenic acid levels are minimal (Giordano et al., 2019).

At the population level, the composition and concentration of phenolic compounds in *C. alba* vary greatly, influenced by geographic location. This pattern has been observed in two studies on *C. alba*. In one study, seven populations were analyzed, showing variation in GAE/g concentrations ranging from 9.83 (±0.05) to 29.85 (±4.39), depending on the season, with the highest levels found in Tiltil, Región Metropolitana, Chile, during both winter and spring (Peña-Rojas et al., 2021). Differences in bark composition from the localities of Cuesta La Dormida and María Pinto, Región Metropolitana, Chile, were noted for the presence of chlorogenic acid, catechin, quercetin, epicatechin, and procyanidins (B1, B2, and C1). Regarding alkaloid content, significant differences were observed in tree bark from three central locations for reticuline, laurotetanine, and *N*-methyllaurotetanine, along with smaller amounts of boldine and laurolitsine (Giordano et al., 2019). Variations were also detected in fruit polyphenols, particularly in the concentrations of 5-caffeoylquinic acid, 3-caffeoylquinic acid, and (−)-epicatechin (Antileo-Laurie et al., 2023).

Variability in the harvesting season is a key factor in the sustainable management of secondary metabolites, helping determine the optimal time for phytochemical extraction. For example, in *C. alba*, significant differences in total flavonoid content (TFC) and total phenolic content (TPC) values have been observed in young branches and leaves across different periods over 1 year (Peña-Rojas et al., 2021) and over 2 years (Giordano et al., 2019). However, the analysis primarily focuses on three compounds found in the branches of *C. alba*: protocatechuic acid, caffeic acid, and vanillic acid. The concentrations of compounds like chlorogenic, caffeic, ferulic, and protocatechuic acids in *C. alba* are notably higher during spring, especially in the localities of Casablanca (Region de Valparaiso, Chile) and Til Til (Region Metropolitana, Chile), and tend to decrease or become undetectable in summer (Peña-Rojas et al., 2021). During spring, levels of catechin, epicatechin, procyanidins, and quercitrin are elevated, while the most significant change was observed with isorhamnetin, which shows very high values in autumn and summer (Giordano et al., 2019).

The concentration of polyphenols in the leaves of cultivated *C. alba* individuals can affect the levels of specific polyphenols, increasing catechin, epicatechin, quercetin, quercitrin, and procyanidins (B1, B2, and C1). Similarly, light exposure also influences the production of some alkaloids in the leaves. In areas with low light (80% shade), higher levels of higenamine, *N*-methylcoclaurine, *N*-methyllaurotetanine, and isocoridine were observed. Conversely, in areas with more light (40% shade), higher concentrations of laurolitsine, boldine, and coclaurine were observed, with no significant differences in reticuline and laurotetanine content (Giordano et al., 2019).

The allelopathic effects of two invasive species, *Ulex europaeus* L. (UEL) and *Teline monspessulana* L. (TEL) [Fabaceae], on the production of phenolic compounds in *C. alba* seedlings were recently examined. Both UEL and TEL extracts significantly inhibited the growth of *C. alba* seedlings, evidenced by shorter stems and roots, fewer leaves, and reduced aerial dry mass (Rodríguez-Cerda et al., 2023). Treatment with TEL notably increased total anthocyanin content in leaves, whereas UEL had no significant effect. Concerning TPC levels, the extract from the aerial part of UEL generally decreased them, whereas the total extract from TEL did not affect overall levels. However, both extracts had different effects on the concentrations of 3,4-dimethylbenzyl alcohol and two specific phenols: vanillin and chlorogenic acid (Rodríguez-Cerda et al., 2023). At low concentrations, both extracts reduced the leaves' antioxidant capacity, with UEL showing greater potency. Additionally, UEL decreased antioxidant capacity as measured by the ABTS assay, whereas TEL showed no significant effect (Rodríguez-Cerda et al., 2023).

## 9 Regulatory status of the medicinal use of *Cryptocarya alba*

In Chile, plant species or their parts, whether processed or not, intended for medicinal or pharmaceutical use, are regulated by the national control system for human-use pharmaceutical products. In the case of plants for medicinal use, both plant markers, which are defined chemical constituents independent of their therapeutic activity and allow for the calculation of the plant’s active principles in the final product, and active principles, which have a specific pharmacological effect or acquire one upon administration to the organism, are fundamental. This regulation covers all plant preparations made from biomass, including extracts, tinctures, juices, oils (both fatty and essential oils), resins, and other products resulting from a specific process, but excludes their chemically defined isolated constituents.

According to Article 8 of Supreme Decree No. 3 of 2010 (Ministerio de Salud de Chile, 2011), the National Institute of Public Health (ISP) is responsible for establishing, through a well-reasoned resolution, the appropriate control regime for products claiming or possessing specific properties. To date, no phytopharmaceutical product containing *C. alba* has been registered for commercial use. Traditional Herbal Medicines (THMs) are classified as pharmaceutical products. However, despite their cultural significance and medicinal uses, peumo was not included in Exempt Resolution No. 522 of 2007 (Ministerio de Salud de Chile, 2007), issued by the Chilean Ministry of Health (MINSAL), which did not list it among the 50 THMs created by MINSAL, nor was it included in Exempt Resolution 190 of 2008 of MINSAL (Ministerio de Salud de Chile, 2008), which expanded that list. Furthermore, its medicinal use has not been authorized through the List of THMs, as approved by Technical Standard No. 133 and Exempt Decree No. 25 of 2012 of MINSAL (Ministerio de Salud de Chile, 2012). Consequently, its traditional therapeutic uses for symptomatic relief have not been officially recognized. Despite efforts by MINSAL since 1991, the regulation of medicinal plants in Chile has been slow. The limited information and lack of robust analytical methods create a regulatory gap that jeopardizes safety. It is essential to increase research, adopt quality control methods, and educate consumers. Only then can the safe and effective use of medicinal plants for public health be ensured (Fuentes-Barros et al., 2025).

## 10 Pharmacokinetic properties

The Administration, Distribution, Metabolism, and Excretion (ADME) properties of the secondary metabolites present in *C. alba*, calculated by the SwissADME platform, are summarized in Supplementary Table S1 (Supplementary Material).

One of the most well-known traditional uses of *C. alba* is the preparation of infusions to treat liver diseases (Muñoz Schick and Barrera, 1981). In this context, the alkaloids found in *C. alba* show solubility levels ranging from soluble to moderately soluble in water, supporting their effective presence in traditional infusions.

Regarding their metabolism, calculated pharmacokinetic properties, such as ADME values, suggest that these alkaloids interact with multiple isoforms of the cytochrome P450 (CYP) enzyme system, primarily found in the liver. Notably, all analyzed alkaloids exhibit inhibitory activity against CYP2D6, with selective inhibition of CYP1A2 and CYP3A4 isoforms. In contrast, no inhibition is expected for CYP_2C19_ and CYP_2C9_. This variation in enzyme interactions suggests distinct metabolic pathways that may help mitigate the adverse effects commonly associated with hepatotoxicity. Additionally, Supplementary Figure S1 (Supplementary Material) displays the BOILED-Egg diagram for several alkaloids found in *C. alba* extracts. The placement of these compounds near the boundary between the yolk and the white indicates moderate lipophilicity. Interestingly, all appear as blue dots, suggesting they are P-gp substrates. This feature may support better absorption and distribution, increasing their potential to produce therapeutic effects in the liver.

Another traditional use of *C. alba* involves applying its essential oil topically to treat skin wounds (Bravo et al., 2017). Key components identified in the oil include eucalyptol, beta-phellandrene, and alpha-terpineol, all of which have been shown to have antimicrobial properties (Touma et al., 2020). From an ADME perspective, these three compounds have lipophilicity (iLogP) values between 2.51 and 2.65, while their predicted skin permeability (LogK_p_) ranges from −4.69 to −5.30. When compared to other metabolites in the plant, 85 compounds—about 59% of the total—show similar or better lipophilicity and skin permeability, suggesting many *C. alba* essential oil metabolites could have favorable pharmacokinetic properties for topical use and may also act as antimicrobials. Additionally, Supplementary Figure S2 (Supplementary Material) shows that some compounds in the essential oil are highly lipophilic, as they are located within the yolk region of the BOILED-Egg plot. These compounds are represented by red dots, indicating they are not substrates of P-glycoprotein. This trait is advantageous for topical activity, potentially improving compound retention and effectiveness at the application site. *Cryptocarya alba* is also traditionally used to treat rheumatism by applying poultices (de Moesbach, 1992; Muñoz Schick and Barrera, 1981). Rheumatism involves chronic inflammation, where excessive ROS production is a key factor. Compounds with antioxidant properties, such as polyphenols, can neutralize these oxidants (Mitsi et al., 2025). Given the topical application in traditional medicine, water solubility and skin permeability are key factors affecting local therapeutic effectiveness. Several polyphenols in *C. alba*, including caffeic acid, ferulic acid, gallic acid, protocatechuic acid, isorhamnetin, kaempferol, sexangularetin, peonidin, and petunidin, are classified as soluble or highly soluble in water. Moreover, all these compounds exhibit favorable skin permeability (LogKp > −7.0), suggesting strong potential for transdermal absorption. The BOILED-Egg diagram (Supplementary Figure S3, Supplementary Material) was utilized to assess the ADME-related properties of various polyphenolic compounds found in *C. alba*. Most of these compounds are located within the white region of the plot, suggesting high hydrophilicity and a strong profile for gastrointestinal absorption. Notably, all compounds—except for 36 and 37—are predicted to be non-substrates of P-glycoprotein. This trait could improve their bioavailability and support their potential anti-rheumatic effects through antioxidant mechanisms.

## 11 Future perspectives

### 11.1 Reticuline from *Cryptocarya alba*: a natural source for alkaloid production

Due to their low abundance in nature and the structural complexity that hinders large-scale chemical synthesis, many medicinally important alkaloids are produced by reconstructing and optimizing their biosynthetic pathways (Bali Judicaël Tra et al., 2022; Pyne and Martin, 2022). The alkaloid reticuline is of great interest to chemical and biotechnology laboratories because it is the key “intermediate” in the biosynthesis of most isoquinoline and related alkaloids. Its high production cost has led to an increased search for new natural sources to supply it. As alternatives, innovative methods that utilize reticuline as a precursor to produce drugs such as codeine and morphine through various biotechnological techniques have been published in prestigious journals worldwide (Bali Judicaël Tra et al., 2022; Pyne and Martin, 2022). An example is the production of chelirithrine from (*S*)-reticuline in *Saccharomyces cerevisiae*, achieved through genetic reprogramming (Zhu et al., 2024).

Metabolic engineering and computational enzyme design provide powerful strategies for optimizing the production of valuable compounds, such as reticuline, in microorganisms. With current technology and tools, processes can be significantly improved through bypass pathways, like those predicted by the M-path computational platform (Takenaka et al., 2024). Reticuline production in *E. coli* is limited by the formation of 3,4-dihydroxyphenylacetaldehyde (DHPAA), a crucial precursor. Conventional pathways use enzymes that produce toxic hydrogen peroxide as a byproduct. Conversely, the CYP_79_ enzyme is emerging as an effective alternative, allowing the conversion of arylacetaldoxime to DHPAA without generating this harmful byproduct (Takenaka et al., 2024).

### 11.2 *Cryptocarya alba* for the green synthesis of nanomaterials

Nanotechnology, driven by advances in materials science and technology, has emerged as one of the most promising fields of the 21st century, offering significant potential for enhancing industrial products and processes. In this context, a quick, eco-friendly, and affordable method for synthesizing silver nanoparticles (AgNPs) has been developed using *C. alba* leaf extracts (Recio-Sánchez et al., 2019). This method enables control over AgNPs' properties by adjusting the concentrations of silver nitrate (AgNO_3_) and *C. alba* extract, resulting in crystalline, spherical AgNPs with an average diameter of 3.5 nm. These AgNPs, synthesized from *C. alba* leaf extract, have proven effective catalysts for degrading the methylene blue dye in industrial settings, highlighting the green synthesis of nanomaterials (Recio-Sánchez et al., 2019). The environmentally friendly synthesis of magnetite nanoparticles (Fe_3_O_4_ NPs) using *C. alba* leaf extract has also been recently reported, employing an ecological, rapid, and low-cost method that opens new opportunities for water bioremediation with nanomaterials. These nanoparticles, with an average size of 12–15 nm and spherical shape, showed promising capabilities for removing contaminants from wastewater, significantly reducing chemical oxygen demand, phosphates, and nitrates (Alarcón-Aravena et al., 2022).

## 12 Concluding remarks

*Cryptocarya alba* exemplifies the rich intersection of ecological, cultural, and phytochemical significance. Its traditional uses—ranging from medicinal applications for liver diseases, rheumatism, and infections to its role as a food source for prehistoric populations—underscore its value as biocultural heritage.

One of *C. alba*’s strengths is its ability to integrate ancestral practices with local knowledge systems, thereby complementing, contextualizing, and strengthening the growing body of scientific research on its bioactive potential. Recent research has shown that the species exhibits antioxidant, anti-inflammatory, vasoprotective, and antimutagenic effects, as well as insecticidal and antifungal properties, highlighting its potential for pharmaceutical and functional food development.

Among the limitations of studies on *C. alba* is the difficulty in standardizing the extracts due to the wide variability in its phytochemical composition. This, combined with the lack of toxicological studies on its extracts or isolated compounds, and the absence of preclinical and clinical studies in humans, limits its safe and effective application.

To unlock its full potential while promoting sustainability, future research and public policies should focus on clarifying its mechanisms of action, supporting its integration into conservation-based cultivation systems, and acknowledging its cultural importance within Chile’s threatened sclerophyllous ecosystems (Fuentes-Barros et al., 2025; Mykhailenko et al., 2025). This approach will help preserve both the biological and cultural legacy of *C. alba* for future generations.

## Glossary

ABTS: 2,2′-azino-bis-(3-ethylbenzothiazoline-6-sulfononic acid) diammonium salt
Ach: Acetylcholine
ACN: Anthocyanins
AD: Anno Domini
ADME: Administration Distribution Metabolism and Excretion
AFDF: Alkaloidal-rich fraction
AgNPs: silver nanoparticles
Akt: Protein kinase B
AMPK: AMP-activated protein kinase
AMs: Acyclic monoterpenes
ASs: Acyclic sesquiterpenes
B: Bark
BAs: Benzoic acid derivatives
BMs: Bicyclic monoterpenes
BSs: Bicyclic sesquiterpenes
CAE: Chlorogenic acid equivalents
CAs: Cinnamic acid derivatives
CATs: Catechins
CGA: Chlorogenic acid
CNS: central nervous system
Co-I: co-injection
DPPH: 2,2-Diphenyl-1-picrylhydrazyl
CYP: Cytochrome P450
DW: Dry weight
DZP: Diazepam
EC: Epicatechin
ECP: Early Ceramic Period
EMS: Ethyl methanesulfonate
EOs: essential oils
F: Fruits
FRAP: Ferric reducing antioxidant potential
Fs: Flavonoids
FW: Fresh weight
G-: Gram-negative
G+: Gram-positive
GAE: Gallic acid equivalents
GC-MS: Gas chromatography-mass spectrometry
HPLC-DAD-MS: High-performance liquid chromatography−diode-array detection−mass spectrometry
HR: High resolution
**IC**_**50**_: 50% Inhibitory concentration
IGF: Insulin-like growth factor
IL-6: Interleukin-6
i.p.: Intraperitoneally
ISP: National Institute of Public Health
JAK2: Janus kinase
L: leaves
**LC**_**50**_: Median lethal concentration
**LD**_**50**_: Median lethal dose
LEP: Late Early Period
LogKp: Logarithm of skin permeability constant
logP: Lipophilicty
LPS: Lipopolysaccharide
MCs: Miscellaneous compounds
MIC: Minimum inhibitory concentration
MINSAL: Chilean Ministry of Health
MMs: Monocyclic monoterpenes
MPCs: Miscellaneous phenolic compounds
MSs: Monocyclic sesquiterpenes
mTOR: Mammalian target of rapamycin
NF-κB: Nuclear factor kappa-light-chain-enhancer of activated B cells
NMR: Nuclear magnetic resonance
Nrf2: Nuclear factor erythroid 2-related factor 2; NO nitric oxide
ORAC: Oxygen radical absorbance capacity
P38 MAPK: p38 subgroup of mitogen-activated protein kinases
pD2: Agonist potency
PI3K: Phosphatidylinositol 3-kinase
PCNs: Procyanidin
PCR: Polymerase chain reaction
QE: Quercetin equivalents
R: Roots
RI: Retention index
RNS: Reactive nitrogen species
ROS: Reactive oxygen species
RS: Reference standard
SOCS3: Suppressor of cytokine signalling 3
STAT3: Signal transducer and activator of transcription 3
TE: Trolox equivalents
TEL: *Teline monspessulana* L.
TFC: Total flavonoid content
THM: Traditional herbal medicines
TNF-α: Tumor necrosis factor-alpha
TPC: Total phenolic content
TSs: Tricyclic sesquiterpenes
UEL: *Ulex europaeus* L.
UHPLC/MS-MS: Ultra-high performance liquid chromatography–tandem mass spectrometry
XO: Xanthine oxidase
W: wood
