# Supplementary material for: Cryptocarya alba (Peumo): an endemic Chilean tree with phytochemicals with bioactive potential
Source: Front Pharmacol. 2025 Dec 3;16:1665897. doi: 10.3389/fphar.2025.1665897 (PMC12709134; doi:10.3389/fphar.2025.1665897)
Supplement: Supplementary file 1 [file Supplementaryfile1.docx]

**Table S1.** ADME properties of secondary metabolites identified in *Cryptocarya alba*, calculated using the SwissADME platform (Daina et al., 2017).

| **n°** | **MW^1^** | **HBA^2^** | **HBD^3^** | **iLOGP^4^** | **ESOL^5^** | **GI^6^** | **BBB^7^** | **P-gp^8^** | **CYP_1A2_^9^** | **CYP_2C19_^10^** | **CYP_2C9_^11^** | **CYP_2D6_^12^** | **CYP_3A4_^13^** | **Log K^14^** | **Lipinski^15^** |  |
| --- | --- | --- | --- | --- | --- | --- | --- | --- | --- | --- | --- | --- | --- | --- | --- | --- |
|  | **Anthocyanin (ACNs), Catechins (CATs), and Procyanidin (PCNs)** | | | | | | | | | | | | | | | |
| 1 | 287.24 | 6 | 5 | -2.62 | S | High | No | Yes | Yes | No | No | No | No | -7.51 | 0 |  |
| 2 | 785.72 | 18 | 9 | -1.21 | MS | Low | No | Yes | No | No | No | No | No | -10.32 | 3 |  |
| 3 | 301.27 | 6 | 4 | -1.94 | S | High | No | Yes | Yes | No | No | No | No | -6.53 | 0 |  |
| 4 | 317.27 | 7 | 5 | -2.01 | S | High | No | Yes | Yes | No | No | No | No | -6.88 | 0 |  |
| 5 | 290.27 | 6 | 5 | 1.33 | S | High | No | Yes | No | No | No | No | No | -7.82 | 0 |  |
| 6 | 290.27 | 6 | 5 | 1.47 | S | High | No | Yes | No | No | No | No | No | -7.82 | 0 |  |
| 7 | 578.52 | 12 | 10 | 1.27 | MS | Low | No | No | No | No | No | No | Yes | -8.15 | 3 |  |
| 8 | 578.52 | 12 | 10 | 1.35 | MS | Low | No | No | No | No | No | No | Yes | -8.15 | 3 |  |
| 9 | 866.77 | 18 | 15 | 2.5 | PS | Low | No | Yes | No | No | No | No | No | -9.24 | 3 |  |
|  | **Benzoic and Cinnamic Acid derivatives (BAs)** | | | | | | | | | | | | | | | |
| 10 | 170.12 | 5 | 4 | 0.21 | VS | High | No | No | No | No | No | No | Yes | -6.84 | 0 |  |
| 11 | 154.12 | 4 | 3 | 0.66 | VS | High | No | No | No | No | No | No | Yes | -6.42 | 0 |  |
| 12 | 180.16 | 4 | 3 | 0.97 | VS | High | No | No | No | No | No | No | No | -6.58 | 0 |  |
| 13 | 164.16 | 3 | 2 | 0.95 | S | High | Yes | No | No | No | No | No | No | -6.26 | 0 |  |
| 14 | 194.18 | 4 | 2 | 1.62 | S | High | Yes | No | No | No | No | No | No | -6.41 | 0 |  |
| 15 | 354.31 | 9 | 6 | 1.01 | VS | Low | No | No | No | No | No | No | No | -8.76 | 1 |  |
| 16 | 354.31 | 9 | 6 | 0.87 | VS | Low | No | No | No | No | No | No | No | -8.76 | 1 |  |
| 17 | 354.31 | 9 | 6 | 0.21 | VS | Low | No | No | No | No | No | No | No | -8.76 | 1 |  |
| 18 | 382.36 | 9 | 5 | 1.1 | S | Low | No | Yes | No | No | No | No | No | -8.44 | 0 |  |
| 19 | 382.36 | 9 | 5 | 1.72 | S | Low | No | Yes | No | No | No | No | No | -8.44 | 0 |  |
|  | **Flavonoids (Fs)** | | | | | | | | | | | | | | | |
| 20 | 450.39 | 11 | 7 | 1.95 | S | Low | No | Yes | No | No | No | No | No | -9.15 | 2 |  |
| 21 | 316.26 | 7 | 4 | 2.04 | S | High | No | No | Yes | No | No | Yes | Yes | -6.46 | 0 |  |
| 22 | 464.38 | 12 | 8 | 1.45 | S | Low | No | No | No | No | No | No | No | -8.88 | 2 |  |
| 23 | 316.26 | 7 | 4 | 2.35 | S | High | No | No | Yes | No | No | Yes | Yes | -6.9 | 0 |  |
| 24 | 478.4 | 12 | 7 | 2.48 | S | Low | No | Yes | No | No | No | No | No | -8.73 | 2 |  |
| 25 | 478.4 | 12 | 7 | 2.58 | S | Low | No | Yes | No | No | No | No | No | -8.73 | 2 |  |
| 26 | 462.4 | 11 | 6 | 2.32 | S | Low | No | Yes | No | No | No | No | Yes | -8.28 | 2 |  |
| 27 | 464.38 | 12 | 8 | 0.94 | S | Low | No | No | No | No | No | No | No | -8.88 | 2 |  |
| 28 | 286.24 | 6 | 4 | 1.7 | S | High | No | No | Yes | No | No | Yes | Yes | -6.7 | 0 |  |
| 29 | 448.38 | 11 | 7 | 1.44 | S | Low | No | No | No | No | No | No | No | -8.52 | 2 |  |
| 30 | 448.38 | 11 | 7 | 1.29 | S | Low | No | No | No | No | No | No | No | -8.52 | 2 |  |
| 31 | 418.35 | 10 | 6 | 1.9 | S | Low | No | No | No | No | No | No | No | -8.3 | 1 |  |
| 32 | 316.26 | 7 | 4 | 2.04 | S | High | No | No | Yes | No | No | Yes | Yes | -6.46 | 0 |  |
| 33 | 478.4 | 12 | 7 | 1.92 | S | Low | No | Yes | No | No | No | No | No | -8.73 | 2 |  |
| 34 | 318.24 | 8 | 6 | 1.08 | S | Low | No | No | Yes | No | No | No | Yes | -7.4 | 1 |  |
| 35 | 448.38 | 11 | 8 | 1 | S | Low | No | No | No | No | No | No | No | -9.14 | 2 |  |
| 36 | 302.24 | 7 | 5 | 1.63 | S | High | No | No | Yes | No | No | Yes | Yes | -7.05 | 0 |  |
| 37 | 478.36 | 13 | 8 | 0.75 | S | Low | No | Yes | No | No | No | No | No | -8.78 | 2 |  |
| 38 | 448.38 | 11 | 7 | 1.6 | S | Low | No | No | No | No | No | No | No | -8.42 | 2 |  |
| 39 | 506.41 | 13 | 7 | 1.7 | S | Low | No | Yes | No | No | No | No | No | -9.12 | 3 |  |
| 40 | 610.52 | 16 | 10 | 0.46 | S | Low | No | Yes | No | No | No | No | No | -10.26 | 3 |  |
| 41 | 342.3 | 11 | 8 | 0.85 | HS | Low | No | Yes | No | No | No | No | No | -11.02 | 2 |  |
|  | **Miscellaneous Compounds (MCs)** | | | | | | | | | | | | | | | |
| 42 | 314.38 | 4 | 2 | 2.97 | S | High | Yes | No | Yes | No | Yes | No | No | -6.29 | 0 |  |
| 43 | 360.44 | 5 | 1 | 3.57 | S | High | Yes | No | No | No | No | Yes | Yes | -6.6 | 0 |  |
| 44 | 324.5 | 3 | 1 | 4.4 | MS | High | Yes | No | Yes | Yes | Yes | Yes | No | -3.2 | 0 |  |
| 45 | 248.36 | 2 | 2 | 3.27 | S | High | Yes | Yes | Yes | No | No | Yes | No | -5.22 | 0 |  |
| 46 | 246.3 | 3 | 1 | 3 | S | High | Yes | No | Yes | Yes | No | No | No | -5.89 | 0 |  |
| 47 | 264.36 | 3 | 0 | 2.93 | S | High | Yes | No | No | No | Yes | No | No | -5.45 | 0 |  |
| 48 | 360.4 | 6 | 3 | 3.17 | S | High | No | Yes | No | No | No | Yes | No | -6.79 | 0 |  |
| 49 | 512.59 | 6 | 2 | 4.41 | PS | High | No | Yes | No | No | Yes | No | Yes | -5.07 | 1 |  |
|  | **Alkaloids** | | | | | | | | | | | | | | | |
| 50 | 327.37 | 5 | 2 | 2.94 | S | High | Yes | Yes | Yes | No | No | Yes | Yes | -6.37 | 0 |  |
| 51 | 355.43 | 5 | 0 | 3.72 | MS | High | Yes | Yes | No | No | No | Yes | Yes | -6.08 | 0 |  |
| 52 | 341.4 | 5 | 1 | 3.48 | S | High | Yes | Yes | Yes | No | No | Yes | Yes | -6.56 | 0 |  |
| 53 | 327.37 | 5 | 2 | 2.93 | S | High | Yes | Yes | Yes | No | No | Yes | Yes | -6.37 | 0 |  |
| 54 | 327.37 | 5 | 2 | 3.03 | S | High | Yes | Yes | Yes | No | No | Yes | Yes | -6.47 | 0 |  |
| 55 | 341.4 | 5 | 1 | 3.24 | MS | High | Yes | Yes | Yes | No | No | Yes | Yes | -6.22 | 0 |  |
| 56 | 341.4 | 5 | 1 | 3.4 | S | High | Yes | Yes | Yes | No | No | Yes | Yes | -6.32 | 0 |  |
| 57 | 341.4 | 5 | 1 | 3.24 | MS | High | Yes | Yes | Yes | No | No | Yes | Yes | -6.22 | 0 |  |
| 58 | 385.34 | 4 | 3 | 2.52 | S | High | Yes | Yes | No | No | No | Yes | No | -6.21 | 0 |  |
| 59 | 299.36 | 4 | 2 | 2.7 | S | High | Yes | Yes | No | No | No | Yes | No | -5.97 | 0 |  |
| 60 | 271.31 | 4 | 4 | 1.84 | S | High | No | Yes | No | No | No | Yes | No | -6.36 | 0 |  |
| 61 | 315.36 | 5 | 3 | 2.98 | S | High | Yes | Yes | No | No | No | Yes | Yes | -6.41 | 0 |  |
| 62 | 329.39 | 5 | 2 | 3.07 | S | High | Yes | Yes | No | No | No | Yes | No | -6.17 | 0 |  |
| 63 | 315.41 | 4 | 1 | 3.12 | S | High | Yes | Yes | No | No | No | Yes | No | -6.51 | 0 |  |
| 64 | 315.41 | 4 | 1 | 3.25 | S | High | Yes | Yes | No | No | No | Yes | No | -6.55 | 0 |  |
|  | **Acyclic Monoterpenes (AMs)** | | | | | | | | | | | | | | | |
| 65 | 154.25 | 1 | 0 | 2.49 | S | High | Yes | No | No | No | No | No | No | -4.52 | 0 |  |
| 66 | 152.23 | 1 | 0 | 2.51 | S | High | Yes | No | No | No | No | No | No | -5.08 | 0 |  |
| 67 | 156.27 | 1 | 1 | 2.72 | S | High | Yes | No | No | No | No | No | No | -4.48 | 0 |  |
| 68 | 154.25 | 1 | 1 | 2.52 | S | High | Yes | No | No | No | No | No | No | -4.71 | 0 |  |
| 69 | 154.25 | 1 | 1 | 2.7 | S | High | Yes | No | No | No | No | No | No | -5.13 | 0 |  |
| 70 | 196.29 | 2 | 0 | 3.08 | S | High | Yes | No | No | No | No | No | No | -4.71 | 0 |  |
| 71 | 182.26 | 2 | 0 | 2.54 | S | High | Yes | No | No | No | No | No | No | -5.05 | 0 |  |
| 72 | 136.23 | 0 | 0 | 2.89 | S | Low | Yes | No | No | No | No | No | No | -4.17 | 0 |  |
| 73 | 154.25 | 1 | 1 | 2.75 | S | High | Yes | No | No | No | No | No | No | -4.71 | 0 |  |
| 74 | 152.23 | 1 | 0 | 2.41 | S | High | Yes | No | No | No | No | No | No | -4.91 | 0 |  |
| 75 | 136.23 | 0 | 0 | 2.89 | S | Low | Yes | No | No | No | No | No | No | -4.17 | 0 |  |
| 76 | 136.23 | 0 | 0 | 2.8 | S | Low | Yes | No | No | No | No | No | No | -4.11 | 0 |  |
| 77 | 136.23 | 0 | 0 | 2.91 | S | Low | Yes | No | No | No | No | No | No | -4.11 | 0 |  |
| 78 | 136.23 | 0 | 0 | 2.94 | S | Low | Yes | No | No | No | No | No | No | -4.21 | 0 |  |
|  | **Monocyclic monoterpenes (MMs)** | | | | | | | | | | | | | | | |
| 79 | 148.2 | 1 | 0 | 2.03 | S | High | Yes | No | Yes | No | No | No | No | -5.52 | 0 |  |
| 80 | 134.22 | 0 | 0 | 2.45 | S | Low | Yes | No | No | No | No | Yes | No | -4.36 | 1 |  |
| 81 | 134.22 | 0 | 0 | 2.52 | S | Low | Yes | No | No | No | No | Yes | No | -3.92 | 1 |  |
| 82 | 134.22 | 0 | 0 | 2.51 | S | Low | Yes | No | No | No | No | Yes | No | -4.21 | 1 |  |
| 83 | 150.22 | 1 | 1 | 2.17 | S | High | Yes | No | Yes | No | No | No | No | -5.8 | 0 |  |
| 84 | 134.22 | 0 | 0 | 2.43 | S | Low | Yes | No | No | No | No | Yes | No | -4.01 | 1 |  |
| 85 | 150.22 | 1 | 1 | 2.32 | S | High | Yes | No | Yes | No | No | No | No | -4.87 | 0 |  |
| 86 | 136.23 | 0 | 0 | 2.72 | S | Low | Yes | No | No | No | Yes | No | No | -3.89 | 0 |  |
| 87 | 136.23 | 0 | 0 | 2.72 | S | Low | Yes | No | No | No | Yes | No | No | -3.89 | 0 |  |
| 88 | 154.25 | 1 | 1 | 2.57 | S | High | Yes | No | No | No | No | No | No | -5.73 | 0 |  |
| 89 | 154.25 | 1 | 1 | 2.4 | S | High | Yes | No | No | No | No | No | No | -5.73 | 0 |  |
| 90 | 122.21 | 0 | 0 | 2.48 | S | Low | Yes | No | No | No | No | No | No | -4.79 | 0 |  |
| 91 | 154.25 | 1 | 1 | 2.41 | S | High | Yes | No | No | No | No | No | No | -5.47 | 0 |  |
| 92 | 136.23 | 0 | 0 | 2.65 | S | Low | Yes | No | No | No | No | No | No | -4.69 | 0 |  |
| 93 | 136.23 | 0 | 0 | 2.72 | S | Low | Yes | No | No | No | No | No | No | -5.18 | 0 |  |
| 94 | 154.25 | 1 | 1 | 2.51 | S | High | Yes | No | No | No | No | No | No | -4.83 | 0 |  |
| 95 | 154.25 | 1 | 1 | 2.51 | S | High | Yes | No | No | No | No | No | No | -4.93 | 0 |  |
| 96 | 154.25 | 1 | 1 | 2.47 | VS | High | Yes | No | No | No | No | No | No | -5.84 | 0 |  |
| 97 | 154.25 | 1 | 1 | 2.51 | S | High | Yes | No | No | No | No | No | No | -4.93 | 0 |  |
| 98 | 154.25 | 1 | 1 | 2.51 | S | High | Yes | No | No | No | No | No | No | -4.83 | 0 |  |
| 99 | 136.23 | 0 | 0 | 2.71 | S | Low | Yes | No | No | No | Yes | No | No | -3.96 | 0 |  |
| 100 | 156.27 | 1 | 1 | 2.55 | S | High | Yes | No | No | No | No | No | No | -4.84 | 0 |  |
| 101 | 154.25 | 1 | 1 | 2.41 | S | High | Yes | No | No | No | No | No | No | -5.45 | 0 |  |
| 102 | 154.25 | 1 | 1 | 2.41 | S | High | Yes | No | No | No | No | No | No | -5.45 | 0 |  |
| 103 | 136.23 | 0 | 0 | 2.73 | S | Low | Yes | No | No | No | No | No | No | -3.94 | 0 |  |
| 104 | 134.22 | 0 | 0 | 2.58 | S | Low | Yes | No | No | No | No | No | No | -4.97 | 0 |  |
| 105 | 136.23 | 0 | 0 | 2.64 | S | Low | Yes | No | No | No | No | No | No | -4.85 | 0 |  |
| 106 | 136.23 | 0 | 0 | 2.7 | S | Low | Yes | No | No | No | No | No | No | -4.11 | 0 |  |
| 107 | 136.23 | 0 | 0 | 2.7 | S | Low | Yes | No | No | No | No | No | No | -5.09 | 0 |  |
|  | **Bicyclic Monoterpenes (BMs)** | | | | | | | | | | | | | | | |
| 108 | 154.25 | 1 | 1 | 2.33 | S | High | Yes | No | No | No | No | No | No | -5.31 | 0 |  |
| 109 | 196.29 | 2 | 0 | 2.5 | S | High | Yes | No | No | No | Yes | No | No | -4.44 | 0 |  |
| 110 | 154.25 | 1 | 1 | 2.27 | S | High | Yes | No | No | No | No | No | No | -5.31 | 0 |  |
| 111 | 196.29 | 2 | 0 | 2.56 | S | High | Yes | No | No | No | Yes | No | No | -4.44 | 0 |  |
| 112 | 136.23 | 0 | 0 | 2.58 | S | Low | Yes | No | No | No | Yes | No | No | -4.13 | 1 |  |
| 113 | 154.25 | 1 | 1 | 2.42 | S | High | Yes | No | No | No | No | No | No | -4.99 | 0 |  |
| 114 | 152.23 | 1 | 0 | 2.21 | S | High | Yes | No | No | No | No | No | No | -4.73 | 0 |  |
| 115 | 136.23 | 0 | 0 | 2.59 | S | Low | Yes | No | No | No | Yes | No | No | -4.18 | 1 |  |
| 116 | 152.23 | 1 | 1 | 2.12 | VS | High | Yes | No | No | No | No | No | No | -5.96 | 0 |  |
| 117 | 152.23 | 1 | 1 | 2.3 | VS | High | Yes | No | No | No | No | No | No | -5.96 | 0 |  |
| 118 | 150.22 | 1 | 0 | 2.15 | S | High | Yes | No | No | No | No | No | No | -5.68 | 0 |  |
| 119 | 150.22 | 1 | 0 | 2.08 | S | High | Yes | No | No | No | No | No | No | -5.1 | 0 |  |
| 120 | 152.23 | 1 | 1 | 2.34 | S | High | Yes | No | No | No | No | No | No | -4.94 | 0 |  |
| 121 | 136.23 | 0 | 0 | 2.63 | S | Low | Yes | No | No | No | Yes | No | No | -3.95 | 1 |  |
| 122 | 136.23 | 0 | 0 | 2.65 | S | Low | Yes | No | No | No | No | No | No | -4.94 | 1 |  |
| 123 | 136.23 | 0 | 0 | 2.67 | S | Low | Yes | No | No | No | No | No | No | -5.11 | 1 |  |
| 124 | 136.23 | 0 | 0 | 2.63 | S | Low | Yes | No | No | No | Yes | No | No | -4.02 | 1 |  |
| 125 | 154.25 | 1 | 0 | 2.68 | S | High | Yes | No | No | No | No | No | No | -5.13 | 0 |  |
| 126 | 154.25 | 1 | 0 | 2.58 | S | High | Yes | No | No | No | No | No | No | -5.3 | 0 |  |
|  | **Bicyclic Monoterpenes (BMs)** | | | | | | | | | | | | | | | |
| 127 | 204.35 | 0 | 0 | 3.89 | MS | Low | No | No | Yes | No | Yes | No | No | -3.2 | 1 |  |
| 128 | 204.35 | 0 | 0 | 3.86 | MS | Low | No | No | Yes | No | Yes | No | No | -3.27 | 1 |  |
| 129 | 222.37 | 1 | 1 | 3.66 | S | High | Yes | No | Yes | No | Yes | No | No | -4.23 | 0 |  |
| 130 | 222.37 | 1 | 1 | 3.64 | S | High | Yes | No | Yes | No | Yes | No | No | -4.23 | 0 |  |
|  | **Monociclic Sesquiterpenes (MSs)** | | | | | | | | | | | | | | | |
| 131 | 204.35 | 0 | 0 | 3.37 | MS | Low | No | No | No | Yes | Yes | No | No | -3.21 | 1 |  |
| 132 | 204.35 | 0 | 0 | 3.43 | MS | Low | No | No | No | Yes | Yes | No | No | -3.8 | 1 |  |
| 133 | 190.32 | 0 | 0 | 3.22 | S | Low | No | No | No | Yes | Yes | No | No | -4.1 | 1 |  |
| 134 | 222.37 | 1 | 1 | 3.2 | S | High | Yes | No | No | No | Yes | No | No | -4.53 | 0 |  |
| 135 | 204.35 | 0 | 0 | 3.67 | MS | Low | No | No | No | No | Yes | No | No | -2.98 | 1 |  |
| 136 | 204.35 | 0 | 0 | 3.31 | MS | Low | No | No | No | No | Yes | No | No | -4.18 | 1 |  |
| 137 | 204.35 | 0 | 0 | 3.29 | S | Low | No | No | No | No | Yes | No | No | -4.32 | 0 |  |
|  | **Bicyclic Sesquiterpenes (BSs)** | | | | | | | | | | | | | | | |
| 138 | 204.35 | 0 | 0 | 3.31 | MS | Low | No | No | No | No | Yes | No | No | -3.38 | 1 |  |
| 139 | 204.35 | 0 | 0 | 3.26 | MS | Low | No | No | No | No | Yes | No | No | -3.45 | 1 |  |
| 140 | 204.35 | 0 | 0 | 3.36 | S | Low | No | No | No | Yes | Yes | No | No | -4.65 | 1 |  |
| 141 | 204.35 | 0 | 0 | 3.39 | S | Low | No | No | No | Yes | Yes | No | No | -4.65 | 1 |  |
| 142 | 204.35 | 0 | 0 | 3.33 | S | Low | No | No | No | Yes | Yes | No | No | -4.65 | 1 |  |
| 143 | 204.35 | 0 | 0 | 3.41 | S | Low | No | No | No | Yes | Yes | No | No | -4.85 | 1 |  |
| 144 | 222.37 | 1 | 1 | 3.15 | S | High | Yes | No | No | Yes | No | No | No | -5.29 | 0 |  |
| 145 | 222.37 | 1 | 1 | 3.24 | S | High | Yes | No | No | Yes | Yes | No | No | -5.03 | 0 |  |
| 146 | 204.35 | 0 | 0 | 3.41 | MS | Low | No | No | No | Yes | Yes | No | No | -2.97 | 1 |  |
| 147 | 204.35 | 0 | 0 | 3.52 | MS | Low | No | No | No | Yes | Yes | No | No | -3.19 | 1 |  |
| 148 | 204.35 | 0 | 0 | 3.29 | MS | Low | No | No | No | Yes | Yes | No | No | -4.21 | 1 |  |
| 149 | 204.35 | 0 | 0 | 3.37 | S | Low | No | No | No | Yes | Yes | No | No | -4.27 | 1 |  |
| 150 | 202.34 | 0 | 0 | 3.16 | MS | Low | No | No | No | No | No | Yes | No | -3.9 | 1 |  |
| 151 | 204.35 | 0 | 0 | 3.25 | S | Low | No | No | No | Yes | Yes | No | No | -4.44 | 1 |  |
| 152 | 222.37 | 1 | 1 | 3.06 | S | High | Yes | No | No | No | Yes | No | No | -5 | 0 |  |
| 153 | 234.33 | 2 | 0 | 2.73 | S | High | Yes | No | No | No | No | Yes | No | -5.34 | 0 |  |
| 154 | 204.35 | 0 | 0 | 3.26 | S | Low | No | No | No | No | Yes | No | No | -4.75 | 1 |  |
|  | **Tricyclic Sesquiterpenes (TSs)** | | | | | | | | | | | | | | | |
| 155 | 204.35 | 0 | 0 | 3.4 | S | Low | Yes | No | Yes | Yes | Yes | No | No | -4.37 | 1 |  |
| 156 | 204.35 | 0 | 0 | 3.38 | MS | Low | Yes | No | Yes | Yes | Yes | No | No | -4.2 | 1 |  |
| 157 | 202.34 | 0 | 0 | 3.18 | S | Low | Yes | No | No | Yes | Yes | No | No | -4.72 | 1 |  |
| 158 | 204.35 | 0 | 0 | 3.26 | S | Low | No | No | No | Yes | Yes | No | No | -4.66 | 1 |  |
| 159 | 204.35 | 0 | 0 | 3.18 | MS | Low | No | No | No | Yes | Yes | No | No | -4.24 | 1 |  |
| 160 | 222.37 | 1 | 1 | 3.02 | S | High | Yes | No | No | Yes | Yes | No | No | -4.62 | 0 |  |
| 161 | 220.35 | 1 | 0 | 3.15 | S | High | Yes | No | No | Yes | Yes | No | No | -5.12 | 0 |  |
| 162 | 204.35 | 0 | 0 | 3.4 | S | Low | Yes | No | Yes | Yes | Yes | No | No | -4.37 | 1 |  |
| 163 | 204.35 | 0 | 0 | 3.2 | S | Low | No | No | No | Yes | Yes | No | No | -4.5 | 1 |  |
| 164 | 222.37 | 1 | 1 | 3.11 | S | High | Yes | No | No | Yes | No | No | No | -5 | 0 |  |
|  | **Miscellaneous Compounds (MCs)** | | | | | | | | | | | | | | | |
| 165 | 172.27 | 0 | 0 | 3.03 | S | Low | Yes | No | No | No | No | No | No | -4.21 | 1 |  |
| 166 | 132.16 | 1 | 0 | 1.65 | S | High | Yes | No | No | No | No | No | No | -5.76 | 0 |  |
| 167 | 208.25 | 3 | 0 | 2.89 | S | High | Yes | No | Yes | No | No | No | No | -5.77 | 0 |  |
| 168 | 148.2 | 1 | 0 | 2.47 | S | High | Yes | No | Yes | No | No | No | No | -4.81 | 0 |  |
| 169 | 164.2 | 2 | 1 | 2.37 | S | High | Yes | No | Yes | No | No | No | No | -5.69 | 0 |  |
| 170 | 178.23 | 2 | 0 | 2.65 | S | High | Yes | No | Yes | No | No | No | No | -5.6 | 0 |  |
| 171 | 136.15 | 2 | 0 | 1.87 | VS | High | Yes | No | No | No | No | No | No | -6.07 | 0 |  |
| 172 | 164.2 | 2 | 0 | 2.36 | S | High | Yes | No | Yes | No | No | No | No | -5.36 | 0 |  |
| 173 | 122.16 | 1 | 1 | 1.7 | VS | High | Yes | No | Yes | No | No | No | No | -6.08 | 0 |  |
| 174 | 192.25 | 2 | 0 | 2.77 | S | High | Yes | No | No | No | No | Yes | No | -5.25 | 0 |  |
| 175 | 206.28 | 2 | 0 | 2.54 | S | High | Yes | No | No | No | No | No | No | -5.03 | 0 |  |
| 176 | 150.13 | 3 | 0 | 1.6 | VS | High | Yes | No | Yes | No | No | No | No | -6.47 | 0 |  |
| 177 | 160.19 | 2 | 0 | 2.46 | S | High | Yes | No | Yes | No | No | No | No | -5.19 | 0 |  |
| 178 | 138.16 | 2 | 0 | 2.07 | S | High | Yes | No | Yes | No | No | No | No | -6.01 | 0 |  |
| 179 | 130.18 | 2 | 1 | 1.79 | VS | High | Yes | No | No | No | No | No | No | -5.38 | 0 |  |
| 180 | 114.19 | 1 | 0 | 2.01 | VS | High | Yes | No | No | No | No | No | No | -5.35 | 0 |  |
| 181 | 116.2 | 1 | 1 | 2.26 | VS | High | Yes | No | No | No | No | No | No | -5.15 | 0 |  |
| 182 | 100.16 | 1 | 0 | 1.77 | VS | High | Yes | No | No | No | No | No | No | -5.65 | 0 |  |
| 183 | 116.16 | 2 | 1 | 1.57 | VS | High | Yes | No | No | No | No | No | No | -5.65 | 0 |  |
| 184 | 102.17 | 1 | 1 | 2.03 | VS | High | Yes | No | No | No | No | No | No | -5.48 | 0 |  |
| 185 | 142.24 | 1 | 0 | 2.56 | S | High | Yes | No | No | No | No | No | No | -4.94 | 0 |  |
| 186 | 140.22 | 1 | 0 | 2.43 | S | High | Yes | No | No | No | No | No | No | -4.92 | 0 |  |
| 187 | 128.21 | 1 | 0 | 2.39 | VS | High | Yes | No | No | No | No | No | No | -5.43 | 0 |  |
| 188 | 130.23 | 1 | 1 | 2.51 | S | High | Yes | No | No | No | No | No | No | -4.96 | 0 |  |
| 189 | 130.23 | 1 | 1 | 2.55 | S | High | Yes | No | No | No | No | No | No | -5.12 | 0 |  |
| 190 | 128.21 | 1 | 1 | 2.33 | VS | High | Yes | No | No | No | No | No | No | -5.27 | 0 |  |
| 191 | 88.15 | 1 | 1 | 1.82 | VS | High | Yes | No | No | No | No | No | No | -5.77 | 0 |  |
| 192 | 80.13 | 1 | 0 | 1.52 | VS | High | Yes | No | No | No | No | No | No | -6.05 | 0 |  |
| 193 | 144.21 | 2 | 0 | 2.57 | VS | High | Yes | No | No | No | No | No | No | -5.63 | 0 |  |
| 194 | 170.25 | 2 | 0 | 2.64 | S | High | Yes | No | No | No | No | No | No | -5.13 | 0 |  |
| 195 | 170.25 | 2 | 0 | 2.93 | S | High | Yes | No | No | No | No | No | No | -5.04 | 0 |  |
| 196 | 88.11 | 2 | 1 | 1.1 | MS | High | Yes | No | No | No | No | No | No | -6.28 | 0 |  |
| 197 | 86.13 | 1 | 0 | 1.49 | VS | High | Yes | No | No | No | No | No | No | -6.12 | 0 |  |
| 198 | 258.4 | 1 | 0 |  |  |  |  |  |  |  |  |  |  |  |  |  |
| 199 | 138.25 | 0 | 0 | 3.2 | S | Low | Yes | No | No | No | No | No | No | -3.98 | 1 |  |
| 200 | 234.33 | 2 | 1 | 2.8 | S | High | Yes | No | No | No | Yes | No | No | -5.59 | 0 |  |
| 201 | 136.23 | 0 | 0 | 2.57 | S | Low | Yes | No | No | No | No | No | No | -5.29 | 0 |  |
| 202 | 316.43 | 3 | 1 | 3.23 | MS | High | Yes | Yes | No | No | No | Yes | No | -5.73 | 0 |  |
| 203 | 224.34 | 2 | 0 | 3.62 | S | High | Yes | No | Yes | No | No | No | No | -4.59 | 0 |  |
| 204 | 166.3 | 0 | 0 | 3.63 | S | Low | No | No | Yes | No | No | No | No | -3.87 | 1 |  |
| 205 | 96.08 | 2 | 0 | 1.03 | VS | High | Yes | No | No | No | No | No | No | -6.6 | 0 |  |
| 206 | 100.16 | 1 | 1 | 1.92 | VS | High | Yes | No | No | No | No | No | No | -5.98 | 0 |  |
| 207 | 330.46 | 3 | 0 | 4.21 | MS | High | Yes | No | Yes | Yes | Yes | Yes | Yes | -4.11 | 0 |  |
| 208 | 126.2 | 1 | 0 | 2.11 | VS | High | Yes | No | No | No | No | No | No | -5.59 | 0 |  |
| 209 | 126.2 | 1 | 0 | 2.17 | VS | High | Yes | No | No | No | No | No | No | -5.73 | 0 |  |
| 210 | 136.23 | 0 | 0 | 2.7 | S | Low | Yes | No | No | No | No | No | No | -5.09 | 0 |  |
| 211 | 220.35 | 1 | 1 | 3.01 | S | High | Yes | No | No | No | Yes | No | No | -4.45 | 0 |  |
| MW^1^: Molecular weigth; HBA^2^: Number of Hydrogen Bonding Acceptor; HBD^3^: Number of Hydrogen Bonding Donor; _i_LOGP^4^: -Log_10_ (Organic/Water); ESOL^5^: Water Solubility Class (VS= Very Soluble, HS= Highly soluble, S= Soluble, MS= Moderately soluble, PS= Poorly soluble); GI^6^: Gastrointestinal Absorption; BBB^7^: blood-brain barrier; P-gp^8^: P-glycoprotein substrate; CYP_1A2_^9^: CYP_1A2_ inhibitor; CYP_2C19_^10^: CYP_2C19_ inhibitor; CYP_2C9_^11^: CYP_2C9_ inhibitor; CYP_2D6_^12^: CYP_2D6_ inhibitor; CYP_3A4_^13^: CYP_3A4_ inhibitor; LogK^14^: Log_10_ (K) representative of Skin permeation; Lipinski^15^: Violation of any Lipinski’s Rules | | | | | | | | | | | | | | | | |


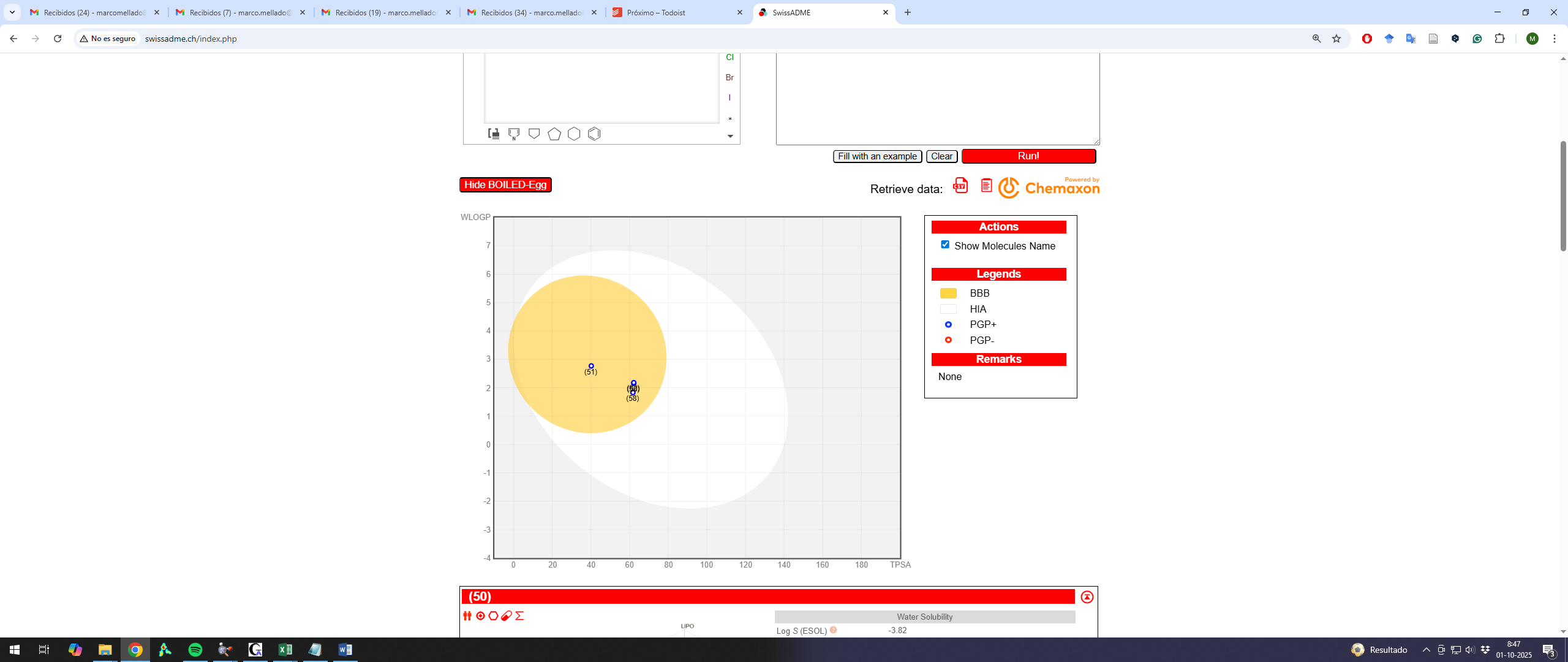


**Figure S1.** Egg boiled scheme of some alkaloids of the *C. alba*. Boldine (**50**), glaucine (**51**), coclaurine (**58**), and reticuline (**62**).


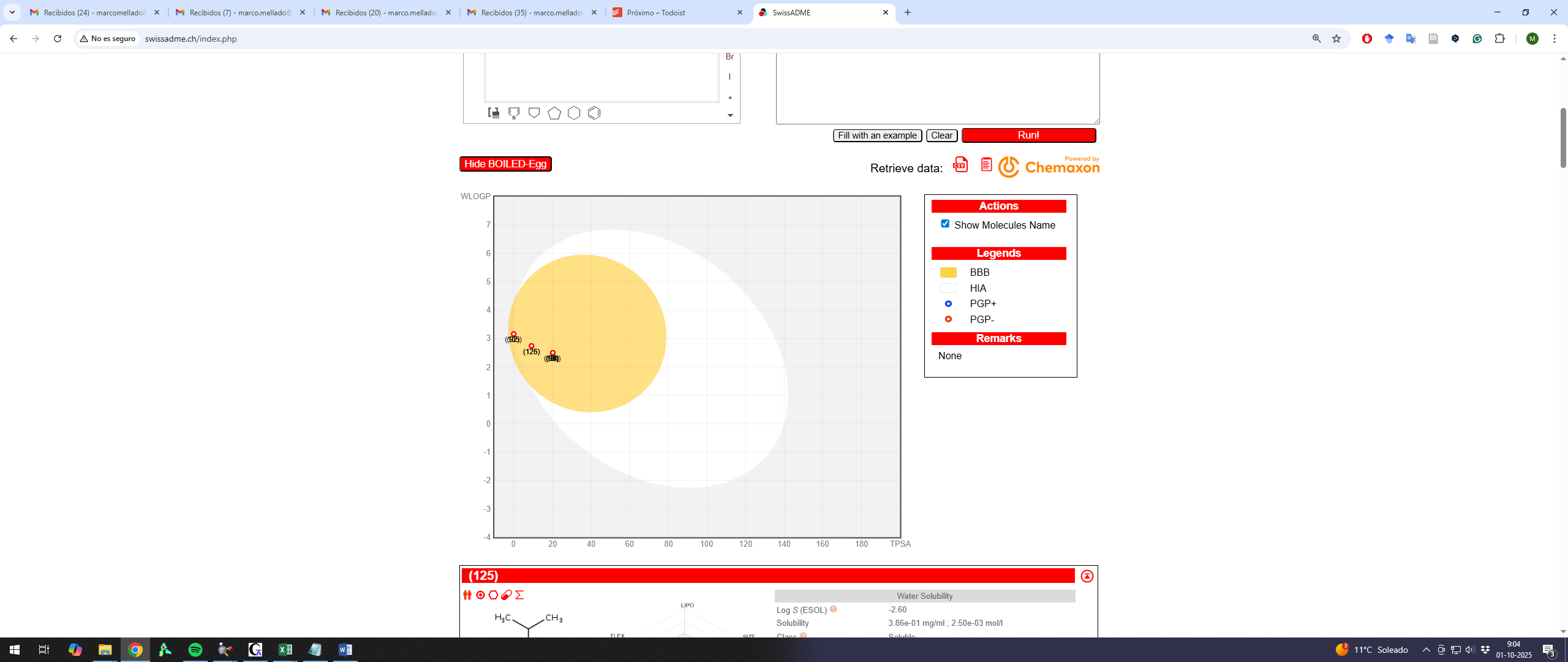


**Figure S2.** Egg boiled scheme of some compounds from the essential oils of the *C. alba*. β-phellandrene (**92**), α-terpineol (**94**), (-)-terpinen-4-ol (**95**), 1-terpineol (**96**), and 4-terpineol (**97**), β-terpineol (**101**), α-phellandrene (**105**), 1,4-cineole (**125**), and 1,8-cineole (**126**).


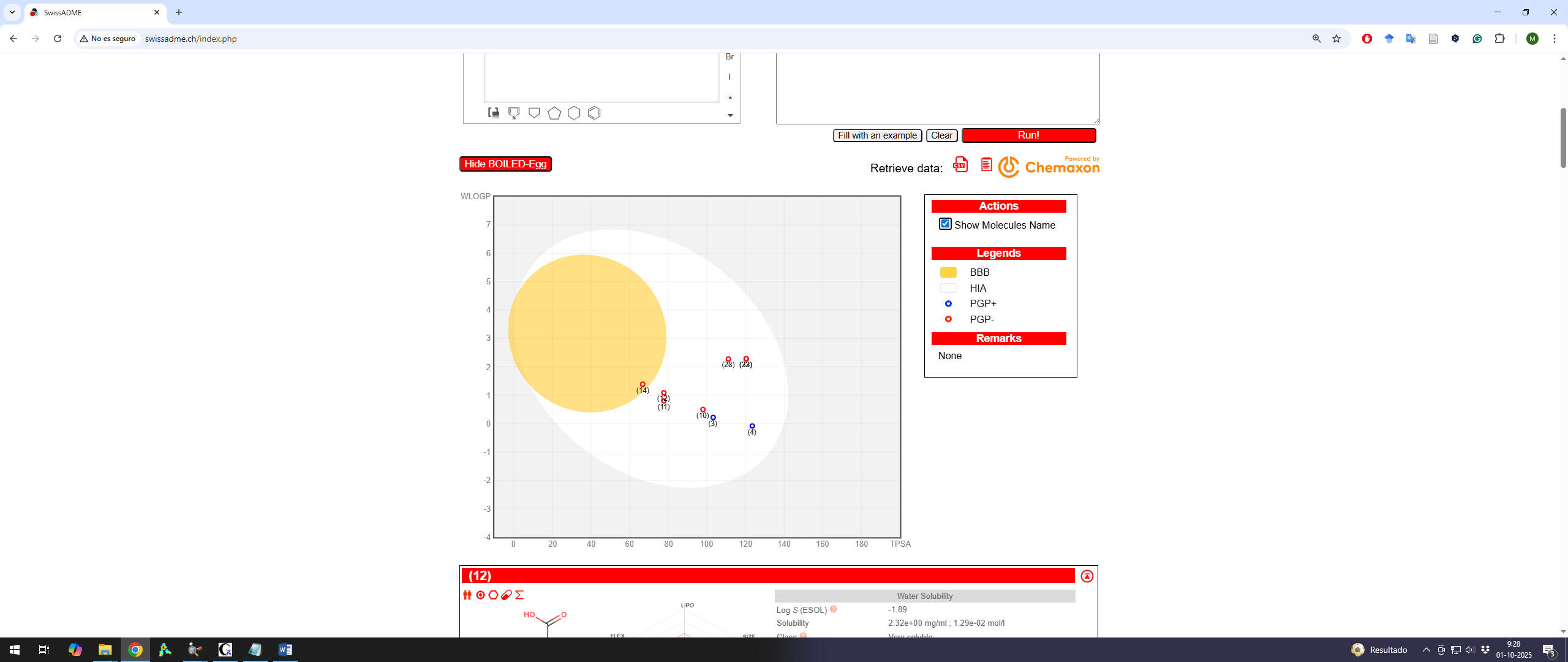


**Figure S3.** Egg boiled scheme of some phenolic compounds of the *C. alba*. Peonidin (**3**), petunidin (**4**), gallic acid (**10**), protocatechuic acid (**11**), caffeic acid (**12**), ferulic acid (**14**), isorhamnetin (**23**), kaempferol (**28**), and sexangularetin (**32**).
